# Supplementary material for: Social determinants and risk factors associated with non-communicable diseases among urban population in Nepal: A comparative study of poor, middle and rich wealth categories of urban population using STEPS survey
Source: PLoS One. 2025 May 14;20(5):e0307622. doi: 10.1371/journal.pone.0307622 (PMC12077703; doi:10.1371/journal.pone.0307622)
Supplement: S1 Table — (DOCX) [file pone.0307622.s001.docx]

Table 1: Wealth Categories in Urban Population

| Three wealth categories | Five wealth Categories | Sample Size |
| --- | --- | --- |
| Urban Poor | Poorest quintile | 1014 |
|  | Second quintile | 680 |
| Urban Middle | Third quintile | 590 |
| Urban Rich | Fourth quintile | 550 |
|  | Fifth quintile | 626 |
