# Supplementary material for: Social determinants and risk factors associated with non-communicable diseases among urban population in Nepal: A comparative study of poor, middle and rich wealth categories of urban population using STEPS survey
Source: PLoS One. 2025 May 14;20(5):e0307622. doi: 10.1371/journal.pone.0307622 (PMC12077703; doi:10.1371/journal.pone.0307622)
Supplement: Attachment — Submitted filename: PONE-D-24-27355_reviewer.pdf [file pone.0307622.s002.pdf]

PLOS ONE

Social determinants and risk factors associated with non-communicable diseases among urban population in Nepal: A comparative study of poor, middle and rich wealth categories of urban population using STEPS survey

--Manuscript Draft--

**Manuscript Number:**	PONE-D-24-27355
**Article Type:**	Research Article
**Full Title:**	Social determinants and risk factors associated with non-communicable diseases among urban population in Nepal: A comparative study of poor, middle and rich wealth categories of urban population using STEPS survey
**Short Title:**	Social determinants and risk factors and non-communicable diseases in urban population
**Corresponding Author:**	Sampurna Kakchapati, Post Doc<br>HERD: Health Research and Social Development Forum<br>Kathmandu, NEPAL
**Keywords:**	Non Communicable disease; Urban; Nepal; Social determinants
**Abstract:**	Background: Non-communicable diseases (NCDs) are a significant public health concern globally, and the burden is disproportionately high among urban populations. This study aims to compare the social determinants, NCD risk, and NCD prevalence among different wealth categories and to determine the factors associated with hypertension, obesity, and diabetes among the urban population of Nepal.<br>Methods: This study used urban population data from STEP wise approach to NCD risk factor surveillance survey of 2019, resulting in a sample of 3460 individuals for inclusion in the analysis. We used bivariate analysis to compare the social determinants, NCD risk and NCD prevalence among urban poor, urban middle and urban rich and multivariate logistic regression to determine the association between social determinants, NCD risks and obesity, hypertension and diabetes among urban population.<br>Results: The study found significant differences in hypertension, obesity and diabetes by gender, ethnicity, education, employment, smoking habits, and cholesterol levels between the three wealth groups. Among the urban poor, low education, unemployment and smoking habits were more prevalent, while high cholesterol was more prevalent among the urban rich. Urban poor from Hilly region (AOR=2.33, 95% CI=1.45-3.75), Bagmati province (AOR=1.82, 95% CI=1.18-2.82) and those who had ever been married (AOR=2.11, 95% CI=1.07-4.13) had higher odds of being overweight and Bagmati province displayed higher odds of obesity (AOR=2.36, 95% CI=1.45-3.75). Being male (AOR=1.68, 95% CI=1.19-2.36), upper caste (AOR=1.78, 95% CI=1.13-2.79) and a homemaker (AOR=3.14, 95% CI = 1.31-7.52) showed significant association with pre-hypertension. Hypertension was associated with being male (AOR=2.23, 95% CI=1.56-3.47), older age (AOR=1.03, 95% CI=1.02-1.04), higher alcohol consumption (AOR=1.58, 95% CI=1.08-2.31), higher cholesterol levels (AOR= 1.03, 95% CI=1.02-1.04) and being overweight (AOR=1.69, 95% CI=1.19-2.39,). Urban population Hilly region (AOR=3.59, 95% CI=1.6-8.08) and Terai region (AOR=5.07, 95% CI=2.44-10.5) had higher odds of pre-diabetes. Obesity was significantly associated with both hypertension (AOR=5.12, 95% CI = 2.95-8.87) and pre-diabetes (AOR=1.82, 95% CI=1.05-3.16). Higher odds of diabetes were associated with originating from the Hilly region (AOR=6.44, 95% CI=3.31-11.10) and Terai regions (AOR = 5.96, 95% CI = 3.12-19.86), Madhesh province (AOR=3.1, 95% CI =1.23-7.83), with university-level education (AOR=3.98, 95% CI=1.39-11.44), and being from a religious minority (AOR= 3.76, 95% CI=1.11-12.73).<br>Conclusion: The findings indicate significant disparities in education, employment, and lifestyle habits across wealth groups; urban poor lacked education and employment. Factors such as ecological region, province and age were associated with an increased risk of various health conditions such as being overweight, pre-hypertension, pre-diabetes, and diabetes. Improved health outcomes among urban populations interventions targeting increased access to education, additional investment in specific areas where outcomes are worst, and interventions to improve equitable access to healthcare are needed.

Powered by Editorial Manager® and ProduXion Manager® from Aries Systems Corporation

Order of Authors:	Sampurna Kakchapati, Post Doc
	Raju Neupane
	Kriti Sagar Baral
	Grishu Shrestha
	Deepak Joshi
	Bryony Dawkins
	Tim Ensor
	Helen Elsey
	Sushil Chandra Baral
Additional Information:	
Question	Response
**Financial Disclosure**<br><br>Enter a financial disclosure statement that describes the sources of funding for the work included in this submission. Review the submission guidelines for detailed requirements. View published research articles from *PLOS ONE* for specific examples.<br><br>This statement is required for submission and **will appear in the published article** if the submission is accepted. Please make sure it is accurate.<br><br>**Funded studies**<br>Enter a statement with the following details:<br>• Initials of the authors who received each award<br>• Grant numbers awarded to each author<br>• The full name of each funder<br>• URL of each funder website<br>• Did the sponsors or funders play any role in the study design, data collection and analysis, decision to publish, or preparation of the manuscript?<br><br>Did you receive funding for this work?	The author(s) received no specific funding for this work.
**Competing Interests**<br><br>Use the instructions below to enter a competing interest statement for this submission. On behalf of all authors, disclose any competing interests that	The authors have declared that no competing interests exist.

could be perceived to bias this work—acknowledging all financial support and any other relevant financial or non-financial competing interests.<br><br>This statement is required for submission and **will appear in the published article** if the submission is accepted. Please make sure it is accurate and that any funding sources listed in your Funding Information later in the submission form are also declared in your Financial Disclosure statement.<br><br>View published research articles from *PLOS ONE* for specific examples.<br><br>**NO authors have competing interests**<br><br>Enter: *The authors have declared that no competing interests exist.*<br><br>**Authors with competing interests**<br><br>Enter competing interest details beginning with this statement:<br><br>*I have read the journal's policy and the authors of this manuscript have the following competing interests: [insert competing interests here]*<br><br>* typeset	
**Ethics Statement**<br><br>Enter an ethics statement for this submission. This statement is required if the study involved:<br><br>• Human participants<br>• Human specimens or tissue<br>• Vertebrate animals or cephalopods<br>• Vertebrate embryos or tissues<br>• Field research<br><br>Write "N/A" if the submission does not require an ethics statement.	The data of STEP survey were approved by NHRC. The data were made publicly available after maintaining the anonymity.

General guidance is provided below. Consult the submission guidelines for detailed instructions. **Make sure that all information entered here is included in the Methods section of the manuscript.**<br><br>**Format for specific study types**<br><br>**Human Subject Research (involving human participants and/or tissue)**<br>• Give the name of the institutional review board or ethics committee that approved the study<br>• Include the approval number and/or a statement indicating approval of this research<br>• Indicate the form of consent obtained (written/oral) or the reason that consent was not obtained (e.g. the data were analyzed anonymously)<br><br>**Animal Research (involving vertebrate animals, embryos or tissues)**<br>• Provide the name of the Institutional Animal Care and Use Committee (IACUC) or other relevant ethics board that reviewed the study protocol, and indicate whether they approved this research or granted a formal waiver of ethical approval<br>• Include an approval number if one was obtained<br>• If the study involved *non-human primates*, add *additional details* about animal welfare and steps taken to ameliorate suffering<br>• If anesthesia, euthanasia, or any kind of animal sacrifice is part of the study, include briefly which substances and/or methods were applied<br><br>**Field Research**<br><br>Include the following details if this study involves the collection of plant, animal, or other materials from a natural setting:<br>• Field permit number<br>• Name of the institution or relevant body that granted permission	
**Data Availability**<br><br>Authors are required to make all data	Yes - all data are fully available without restriction

Powered by Editorial Manager® and ProduXion Manager® from Aries Systems Corporation

underlying the findings described fully available, without restriction, and from the time of publication. PLOS allows rare exceptions to address legal and ethical concerns. See the PLOS Data Policy and FAQ for detailed information.<br><br>A Data Availability Statement describing where the data can be found is required at submission. Your answers to this question constitute the Data Availability Statement and **will be published in the article**, if accepted.<br><br>**Important:** Stating 'data available on request from the author' is not sufficient. If your data are only available upon request, select 'No' for the first question and explain your exceptional situation in the text box.<br><br>Do the authors confirm that all data underlying the findings described in their manuscript are fully available without restriction?	
**Describe where the data may be found in full sentences. If you are copying our sample text, replace any instances of XXX with the appropriate details.**<br><br>• If the data are **held or will be held in a public repository**, include URLs, accession numbers or DOIs. If this information will only be available after acceptance, indicate this by ticking the box below. For example: *All XXX files are available from the XXX database (accession number(s) XXX, XXX.)*.<br>• If the data are all contained **within the manuscript and/or Supporting Information files**, enter the following: *All relevant data are within the manuscript and its Supporting Information files.*<br>• If neither of these applies but you are able to provide **details of access elsewhere**, with or without limitations, please do so. For example:<br><br>*Data cannot be shared publicly because of [XXX]. Data are available from the*	The data of STEP survey were approved by NHRC. The data were made publicly available after maintaining the anonymity.

XXX Institutional Data Access / Ethics Committee (contact via XXX) for researchers who meet the criteria for access to confidential data.<br><br>*The data underlying the results presented in the study are available from (include the name of the third party and contact information or URL).*<br>• This text is appropriate if the data are owned by a third party and authors do not have permission to share the data.<br><br>* typeset	
Additional data availability information:	Tick here if the URLs/accession numbers/DOIs will be available only after acceptance of the manuscript for publication so that we can ensure their inclusion before publication.; Tick here if your circumstances are not covered by the questions above and you need the journal's help to make your data available.

Manuscript Click here to access/download;Manuscript;Manuscript.docx

Social determinants and risk factors associated with non-communicable diseases among urban population in Nepal: A comparative study of poor, middle and rich wealth categories of urban population using STEPS survey

Authors

Sampurna Kakchapati[1], Raju Neupane[1], Kriti Sagar Baral[1], Grishu Shrestha[1], Deepak Joshi[1], Bryony Dawkins[2], Tim Ensor[3], Helen Elsey[4], Sushil Chandra Baral[1]

[1]HERD International

[2]Academic Unit of Health Economics, Leeds Institute of Health Sciences, University of Leeds

[3]Nuffield Centre for International Health and Development, Leeds Institute of Health Sciences, University of Leeds

[4]Hull York Medical School, University of York

Social determinants and risk factors associated with non-communicable diseases among urban population in Nepal: A comparative study of poor, middle and rich wealth categories of urban population using STEPS survey

Abstract

Background: Non-communicable diseases (NCDs) are a significant public health concern globally, and the burden is disproportionately high among urban populations. This study aims to compare the social determinants, NCD risk, and NCD prevalence among different wealth categories and to determine the factors associated with hypertension, obesity, and diabetes among the urban population of Nepal.

Methods: This study used urban population data from STEP wise approach to NCD risk factor surveillance survey of 2019, resulting in a sample of 3460 individuals for inclusion in the analysis. We used bivariate analysis to compare the social determinants, NCD risk and NCD prevalence among urban poor, urban middle and urban rich and multivariate logistic regression to determine the association between social determinants, NCD risks and obesity, hypertension and diabetes among urban population.

Results: The study found significant differences in hypertension, obesity and diabetes by gender, ethnicity, education, employment, smoking habits, and cholesterol levels between the three wealth groups. Among the urban poor, low education, unemployment and smoking habits were more prevalent, while high cholesterol was more prevalent among the urban rich. Urban poor from Hilly region (AOR=2.33, 95% CI=1.45-3.75), Bagmati province (AOR=1.82, 95% CI=1.18-2.82) and those who had ever been married (AOR=2.11, 95% CI=1.07-4.13) had higher odds of being overweight and Bagmati province displayed higher odds of obesity (AOR=2.36,

95% CI=1.45-3.75). Being male (AOR=1.68, 95% CI=1.19-2.36), upper caste (AOR=1.78, 95% CI=1.13-2.79) and a homemaker (AOR=3.14, 95% CI = 1.31-7.52) showed significant association with pre-hypertension. Hypertension was associated with being male (AOR=2.23, 95% CI=1.56-3.47), older age (AOR=1.03, 95% CI=1.02-1.04), higher alcohol consumption (AOR=1.58, 95% CI=1.08-2.31), higher cholesterol levels (AOR= 1.03, 95% CI=1.02-1.04) and being overweight (AOR=1.69, 95% CI=1.19-2.39,). Urban population Hilly region (AOR=3.59, 95% CI=1.6-8.08) and Terai region (AOR=5.07, 95% CI=2.44-10.5) had higher odds of pre-diabetes. Obesity was significantly associated with both hypertension (AOR=5.12, 95% CI = 2.95-8.87) and pre-diabetes (AOR=1.82, 95% CI=1.05-3.16). Higher odds of diabetes were associated with originating from the Hilly region (AOR=6.44, 95% CI=3.31-11.10) and Terai regions (AOR = 5.96, 95% CI = 3.12-19.86), Madhesh province (AOR=3.1, 95% CI =1.23-7.83), with university-level education (AOR=3.98, 95% CI=1.39-11.44), and being from a religious minority (AOR= 3.76, 95% CI=1.11-12.73).

Conclusion: The findings indicate significant disparities in education, employment, and lifestyle habits across wealth groups; urban poor lacked education and employment. Factors such as ecological region, province and age were associated with an increased risk of various health conditions such as being overweight, pre-hypertension, pre-diabetes, and diabetes. Improved health outcomes among urban populations interventions targeting increased access to education, additional investment in specific areas where outcomes are worst, and interventions to improve equitable access to healthcare are needed.

Introduction

Non-communicable diseases (NCDs) are now the leading global causes of death. Diseases such as diabetes, cardiovascular diseases, cancer and chronic respiratory diseases are collectively responsible for 74% of worldwide deaths [1]. A total of 41 million people die each year because of NCDs, 17 million of these are under 70 years of age and more than 85% of these premature deaths are in low- and middle-income countries [2]. This disproportionate distribution of deaths in low- and middle-income countries is an issue of equity and social justice [3]. Modifiable behavioral risk factors, such as tobacco use, physical inactivity, unhealthy diet, and harmful use of alcohol, lead to the major key metabolic and physiological risk factors common to most NCDs, including raised blood pressure, overweight/obesity, raised blood glucose, and raised cholesterol [4]. Socio-economic factors are associated with behavioral risk factors for and mortality for NCDs [3]. Exposure to NCD risk factors occur at the places where people live and work. The conditions in which people are born, grow, work, live and age with the ultimate influence of the local surrounding wider forces, systems and structures are social determinants and they are simply termed as "causes of causes" of health inequity. These are produced in society from inequities in wealth, power and resources which are further based on the dimensions of social stratification, socioeconomic status, gender, ethnicity and disabilities [5,6,7].

In Nepal, the impact of urbanisation, economic development, and changing lifestyles has created both opportunities and challenges in the thriving urban environment [8,9]. Urbanisation has paved the path for advancement, but it has also brought about number of health problems that require prompt action [9,10]. Growing urban populations, along with shifting social and environmental dynamics, have given rise to complex web of interconnected health issues that have huge impact on quality of life [8-10]. Rapid urbanization has led to the concentration of vulnerable populations in urban centers, where they face unique socio-economic challenges, including limited access to

healthcare, inadequate sanitation, and substandard living conditions. These circumstances can exacerbate the risk of NCDs among urban populations, making them a particularly important demographic to study [3,6,7]. The association between urbanization and key biological risk factors for non-communicable diseases was evident in the meta-analysis of data from 173 countries between 1980 and 2008 finding urbanisation contributed to an increase in average BMI and cholesterol levels [11].

The dynamics of urban health in Nepal are associated with a complex and interlinked range of wider determinants, as conceptualized by the Commission on Social Determinants of Health (CSDH) [5]. Urban residents face material constraints, limiting access to nutritious food, quality healthcare, and the ability to maintain healthy lifestyles [3-5]. The rising prevalence of processed, fatty foods alongside declining intake of fruits and vegetables reflects a concerning trend in dietary patterns, contributing significantly to the NCD burden [6,8,11]. Furthermore, increasing rates of tobacco, alcohol, and drug use add layers of complexity to public health challenges. Psychosocial factors, including heightened stress levels and inadequate mental health support, particularly among informal settlement dwellers, has lead to a surge in mental health disorders [2,4,8]. The co-influence of socio-economic factors, including gender and wealth disparities, further magnifies health inequities, creating a complex interplay between social determinants and health outcomes.

Moreover, the urban populations remains a neglected population and evidence and interventions regarding social determinants and risk factors of NCDs among urban population are scarce in many low and middle income countries, including Nepal [12-13]. Within cities, there is a lack of studies comparing different wealth strata. This is in part due to the limited data available from informal settlements and urban poor households, who may be missed in survey sampling frames and even within the census in the country [14]. Understanding the intricate relationship between social

determinants and NCDs among the urban population in Nepal is essential for several reasons [12,13,15]. It provides critical insights into the underlying structural factors that drive health disparities within this demographic. By identifying these determinants, targeted interventions and policy measures can be developed to address the root causes of NCD prevalence [12,13,15]. In this context, this study utilized the data of the 2019 STEPS survey to compare the prevalence of risk factors across wealth quintiles and provide analysis of the factors associated with NCDs within the urban population. This study will contribute valuable insights for the prevention, control and reduction of NCDs by providing relevant information on social determinants and risk factors of NCDs among urban population. Ultimately, this study aims to inform effective strategies that mitigate the burden of NCDs and enhance the overall well-being of the urban population in Nepal.

Methods

Study design and sampling size

This study utilized data from the STEP (Stepwise Approach to Surveillance) survey, a comprehensive cross-sectional survey conducted by World Health Organization (WHO) and Nepal Health Research Council (NHRC) to monitor and evaluate NCD risk factors and related health behaviors. The survey was designed to collect data on the prevalence and distribution of risk factors for NCDs, such as tobacco use, alcohol consumption, physical inactivity, and unhealthy diet. The survey employed a multistage, stratified sampling design to ensure representative coverage of the target population. Data were collected through face-to-face interviews and standardized physical measurements conducted by trained field personnel. The survey instrument included structured questionnaires covering a range of demographic, socioeconomic, lifestyle, and health-related variables. Additionally, physical measurements such as blood pressure,

anthropometric indices, and biochemical parameters were collected following standardized protocols.

Study setting

Nepal is a landlocked country in South Asia between India and China. It has a diverse landscape ranging from plains in the south to hills and mountains in the Himalayas. The country is geographically diverse, comprising three main regions: the mountainous region, the hilly region, and the flat plains of the Terai. The mountainous region, including the Himalayas, is sparsely populated due to rugged terrain and harsh climatic conditions. The hilly region is more densely populated and experiences a mix of urban and rural settlements and the Terai region, characterized by fertile plains, has historically been a hub of agriculture and commerce. Nepal is one of the fastest urbanizing countries in Asia with 66% of the population living in urban areas in 2021 compared to just 23% in 2021. The rural population is more concentrated in the Hill (66%) and Mountain regions (16%). Peri-urban population is concentrated in the Terai region (85%) and urban population is concentrated in the Hill region (53%), and mainly in Pokhara Metropolitan City and Kathmandu valley, followed by the Terai region (39%). An estimated 70 percent of Nepalese migrate to the urban areas for employment and education so as to improve their economic condition and the internal migration is high from terai and mountain to hilly region where the urban areas are concentrated. Nepal is divided into 7 provinces and 753 local levels. The highest urban population concentration is in Madhesh province (19.9%), followed by Bagmati province (14.6%) and the lowest in Karnali province (1.2%), which shows an imbalance in the distribution of the urban population [16].

Participants

Data for the study was obtained from STEP Survey [17]. The STEPS Survey 2019 was designed to be representative of the national population and was carried out between February and May 2019. The survey population included the adult population from 15 to 69 years. The survey used a multistage cluster design: At the first stage, 259 wards were selected from the whole country in which there were total 37 primary sampling units (PSUs) in each province. In the second stage, 25 households were selected from each of the PSU out of the listed sampling frame of households in each ward using systematic random sampling [17]. From each of the selected household, one adult member was sampled randomly for participation in the survey using the android tablet. The STEP dataset included about 6,475 eligible individuals, from which we included 3460 individuals from urban areas in our analysis.

Table 1: Variable definitions

Variables	Definitions
Wealth quintile	Wealth quintiles was classified into 5 category- Lowest, Second, Middle, Fourth and Highest Wealth Quintiles.
Rich Category	Highest and fourth wealth quintiles were combined to form the Rich category.
Middle Category	Middle wealth quintile was used in the analysis as the middle category.
Poor Category	Lowest and Second wealth quintiles were combined to form the Poor category.
Ethnicity groups	Defined using categories in the Nepal Demographic Health Survey as<br>Upper caste Groups, Advantaged Janajatis, Disadvantaged groups, Dalits and Religious Minorities
Ecological region	Ecological region in Nepal is divided into Mountain, Hilly and Terai regions
Obesity	Participants with a BMI greater than or equal to 30 kg/m^2, were classified as being obese.
Overweight	Participants with a BMI greater than or equal to 25 kg/m^2, were classified as being overweight.
Current smoker	Participants who had smoked in the past 30 days were considered as current smokers for this survey.
Harmful alcohol consumption	Harmful use means consumption of greater than or equal to 60 gm of pure alcohol on an average day in the past 30 days

Variables	Definitions
Sufficient fruit and vegetable consumption	Participants who ate five or more servings of fruits and vegetables per day
Sufficient physical activity	Participants who participated in more than or equal to 150 minutes of moderate intensity (600 METs) physical activities per week
24 hours Salt Consumption	Participants who had consumed salts within 24 hours estimated for the first time
Pre-hypertension	Participants were classified as having Pre-Hypertension if the average 2nd and 3rd measurement of systolic BP was greater to 140 mmHg, or the average diastolic BP was greater than 90 mmHg, or if they reported to be taking antihypertensive medication.
Hypertension	Participants were classified as having Hypertension if the average 2nd and 3rd measurement of systolic BP was greater or equal to 140 mmHg, or the average diastolic BP was greater than 90 mmHg, or if they reported to be taking antihypertensive medication.
Pre-diabetes	Participants with a fasting blood sugar greater than 99 mg/dl and less than 126 mg/dl
Diabetes	Participants with a fasting blood sugar greater or equal to 126 mg/dl, or those currently taking medications to lower blood sugar, were considered to be Diabetics
Urban Poor	Participants with poor wealth category from urban population
Urban Middle	Participants with middle wealth category from urban population
Urban Rich	Participants with rich wealth category from urban population

Quantitative variables

The primary outcomes of interest in this study were two common NCDs: diabetes and hypertension. The independent variables selected from the study was adapted from WHO Commission on Social Determinants of Health (CSDH). The structural determinants of health include social class (Gender, Marital Status, Ethnicity, Ecological belt and Province) along with socio-economic position (Education, Occupation and Wealth Quintile). Likewise, the intermediary determinants of health include behavior and biological factors such as alcohol and smoking use, fruit and vegetable consumption, 24 hours salt consumption, sufficient physical activity, age and

cholesterol level. Figure 1 shows the conceptual framework with structural determinants, socio economic position, intermediary determinants and outcomes.

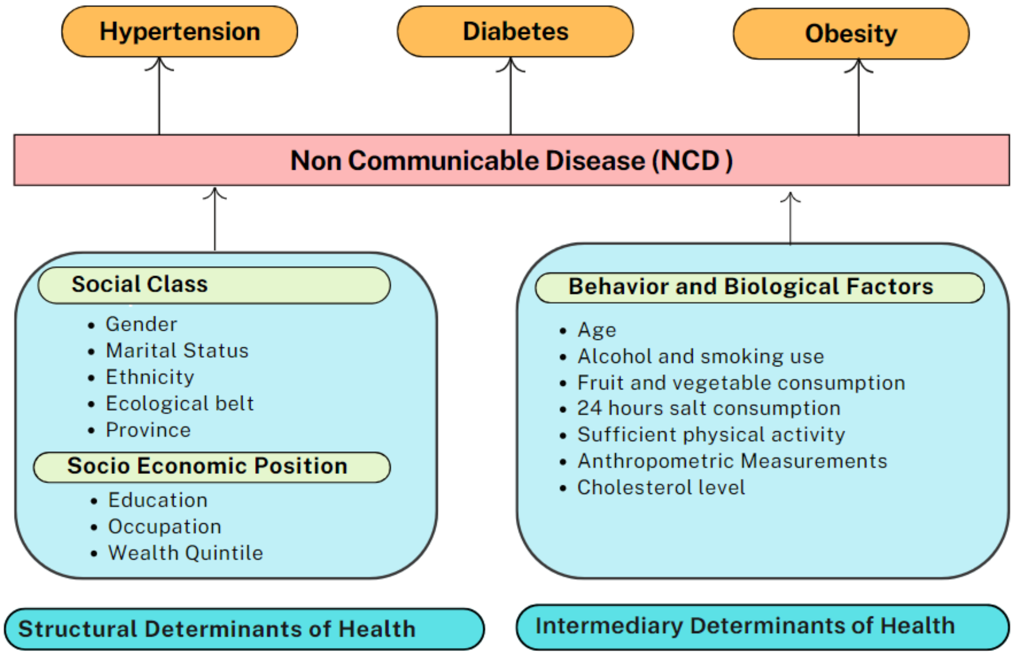

Figure 2: Conceptual Framework for analysis of NCDs and its relationship with social determinants (SDH)

Statistical Methods

Bivariate analyses were conducted to assess the unadjusted associations between the independent variables among urban poor, urban middle and urban rich using chi-square tests for categorical variables and t-tests or ANOVA for continuous variables, as appropriate. We used R version 4.2.0 and RStudio 1314 for statistical analysis. We used "survey" package 15 and performed a weighted analysis to account for the complex survey design of 2020 STEP. We used univariate and multivariate weighted logistic regression analysis to determine the association of the NCDs with independent variables including age, gender, ecological region, province, ethnicity, marital status, education, occupation, smoking, alcohol consumption, physical activity, salt intake. Initially,

explanatory determinants were included in the model one at a time to examine their univariate relationship with the outcome and variables that were significant in the univariate analysis were fitted in the multivariate analysis. Multivariate weighted logistic regression models were then used to identify the most important determinants for each outcome. A p-value of less than 0.05 was used to define statistical significance. Adjusted odds ratios (AORs) as well as their 95% confidence intervals (95% CI) were used to depict the independent relationship between predictors and dependent variables. All statistical analyses for this study were conducted using R program.

Ethics Statement

The data of STEP survey were approved by NHRC. The data were made publicly available after maintaining the anonymity. All data used in this study were fully anonymized before being accessed and identifiers such as patient names, addresses, and contact details were removed to protect patient confidentiality.

Results

Table 1 shows the comparison of risk behaviors that include socio demographic factors, behavioral factors biological factors and NCD among poor, middle and rich wealth categories of urban population . Slightly less than half of the urban population (49%) are in the richest wealth category whereas about one third of urban population are in poorest category (34%). There was a significant association among education status, occupation status, current use of tobacco products and cholesterol level within the three wealth categories of urban population ($p < 0.05$). Urban population with higher levels of education are more prevalent in the rich category, whereas those with little or no formal education are more likely to be poor. Employment is higher in the middle and rich category, while homemakers are more predominant in the poor category. Smokers are

more prevalent in the rich category compared to middle and poor category. There were notable variations in total cholesterol across different economic strata and the urban poor category had higher cholesterol levels compared to middle and rich categories.

Table 1: Comparison of social determinants, NCD risk behaviors and NCD among urban poor, urban middle and urban rich

Characteristics	Wealth Categories in Urban Population				P value
	Urban Rich (n=1694, 49%)	Urban Middle (n=590, 17%)	Urban Poor (n=1176, 34%)	Total (N=3460)	
**Socio Demographic Factors**					
Age					0.229
Median (IQR)	38 (30,51)	37 (28,50.8)	38 (29,49)	38 (29,50)	
Gender					0.063
Female	1120 (50.5)	369 (16.6)	731 (32.9)	2220 (64.2)	
Male	574 (46.3)	221 (17.8)	445 (35.9)	1240 (35.8)	
Ecological Belt					0.352
Mountain	164 (52.9)	51 (16.5)	95 (30.6)	310 (9)	
Hilly	710 (50)	240 (16.9)	471 (33.1)	1421 (41.1)	
Terai	820 (47.4)	299 (17.3)	610 (35.3)	1729 (50)	
Province					0.121
Koshi	246 (51.8)	68 (14.3)	161 (33.9)	475 (13.7)	
Madhesh	267 (45.8)	102 (17.5)	214 (36.7)	583 (16.8)	
Bagmati	279 (48.9)	84 (14.7)	208 (36.4)	571 (16.5)	
Gandaki	231 (47.4)	98 (20.1)	158 (32.4)	487 (14.1)	
Lumbini	210 (49.3)	68 (16)	148 (34.7)	426 (12.3)	
Karnali	216 (52.9)	69 (16.9)	123 (30.1)	408 (11.8)	
Sudurpashchim	245 (48)	101 (19.8)	164 (32.2)	510 (14.7)	
Education Status					**0.029**
No formal education	807 (50.4)	270 (16.9)	525 (32.8)	1602 (46.3)	
Primary	317 (49.5)	123 (19.2)	201 (31.4)	641 (18.5)	
Secondary	493 (45.7)	177 (16.4)	408 (37.8)	1078 (31.2)	
University	77 (55.4)	20 (14.4)	42 (30.2)	139 (4)	
Ethnicity					0.178
Advantaged Janajatis	277 (50.5)	94 (17.2)	177 (32.3)	548 (15.8)	
Dalits	225 (47.8)	97 (20.6)	149 (31.6)	471 (13.6)	
Disadvantaged groups	472 (47.1)	157 (15.7)	373 (37.2)	1002 (29)	
Religious Minorities	65 (53.7)	19 (15.7)	37 (30.6)	121 (3.5)	
Upper caste Groups	655 (49.7)	223 (16.9)	440 (33.4)	1318 (38.1)	
Marital Status					0.166
Unmarried	153 (45)	70 (20.6)	117 (34.4)	340 (10.4)	
Married	1445 (49.3)	487 (16.6)	997 (34)	2929 (89.2)	

Separated	5 (33.3)	2 (13.3)	8 (53.3)	15 (0.5)	
Occupation Status					**0.018**
Unemployed	107 (58.5)	26 (14.2)	50 (27.3)	183 (5.3)	
Students	117 (47)	46 (18.5)	86 (34.5)	249 (7.2)	
Home maker	935 (50.5)	314 (17)	603 (32.6)	1852 (53.6)	
Employed	427 (46.7)	153 (16.7)	335 (36.6)	915 (26.5)	
Others	106 (41.1)	50 (19.4)	102 (39.5)	258 (7.5)	
**Behavioral Factors**					
Current Smoking					**0.003**
No	1358 (47.6)	496 (17.4)	998 (35)	2852 (82.4)	
Yes	336 (55.3)	94 (15.5)	178 (29.3)	608 (17.6)	
Alcohol Consumption					0.694
No	1233 (48.7)	440 (17.4)	859 (33.9)	2532 (73.2)	
Yes	461 (49.7)	150 (16.2)	317 (34.2)	928 (26.8)	
Sufficient fruit and vegetable consumption					0.263
Insufficient	1433 (48.5)	515 (17.4)	1008 (34.1)	2956 (85.4)	
Sufficient	261 (51.8)	75 (14.9)	168 (33.3)	504 (14.6)	
Sufficient physical activity					0.936
Insufficient	289 (49.6)	97 (16.6)	197 (33.8)	583 (16.8)	
Sufficient	1405 (48.8)	493 (17.1)	979 (34)	2877 (83.2)	
24 hours Salt Consumption					0.985
No	355 (49)	122 (16.9)	247 (34.1)	724 (20.9)	
Yes	1339 (49)	468 (17.1)	928 (33.9)	2735 (79.1)	
**Biological Factors**					
Total Cholesterol					**0.004**
Median (IQR)	139 (114,169)	134 (114.2,167)	144 (118,171)	140 (115,169)	
Obesity					0.366
Normal	983 (49.6)	338 (17.1)	660 (33.3)	1981 (58.1)	
Obesity	116 (51.8)	36 (16.1)	72 (32.1)	224 (6.6)	
Overweight	410 (45.8)	153 (17.1)	333 (37.2)	896 (26.3)	
Underweight	160 (51.6)	51 (16.5)	99 (31.9)	310 (9.1)	
Health Outcomes					
Diabetes					0.337
Normal	1205 (49.3)	419 (17.1)	820 (33.6)	2444 (70.6)	
Pre-diabetic	357 (46.5)	137 (17.9)	273 (35.6)	767 (22.2)	
Diabetic	132 (53)	34 (13.7)	83 (33.3)	249 (7.2)	
Hypertension					0.554
Normal	892 (49.7)	300 (16.7)	602 (33.6)	1794 (51.8)	
Pre-Hypertension	347 (46.3)	132 (17.6)	271 (36.1)	750 (21.7)	
Hypertension	455 (49.7)	158 (17.2)	303 (33.1)	916 (26.5)	

Table 2 shows the factors associated with being overweight and obese in multivariate logistic regression among urban population. Disadvantaged ethnic groups had lower odds of being

overweight (AOR=0.52, 95% CI =0.36- 0.78, p=0.001) and lower odds of being obese (AOR=0.45, 95% CI =0.22-0.92, p=0.028) compared to advantaged ethnic groups. Urban Population in the Hilly region (AOR=2.33, 95% CI = 1.45-3.75, p = 0.001) had higher odds of being overweight compared to the mountain region. Likewise, urban population from Bagmati province had higher odds of obesity compared to Koshi province (AOR=2.36, 95% CI = 1.45-3.75, p = 0.001). Similarly, urban population in Bagmati province (AOR = 1.82, 95% CI = 1.18-2.82, p = 0.007) had higher odds of being overweight whereas as urban population in Karnali province (AOR = 0.36, 95% CI = 0.22-0.58, p = 0.001 and Sudurpaschim province (AOR=0.42, 95%CI=0.26-0.66, p=0.001) had lower odds of being overweight compared to Koshi province. Participants who had ever been married had higher odds of being overweight (AOR = 2.11, 95% CI = 1.07- 4.13, p = 0.03) compared to urban population who had never married. Those with high cholesterol had higher odds of being overweight (AOR = 1.01 95% CI = 1.00-1.02, $p < 0.001$) and obesity (AOR = 1.01, 95% CI = 1.01-1.02, $p < 0.001$) compared to people with normal cholesterol levels. There was no significant difference in obesity and overweight within wealth quintiles and between males and females.

Table 2: Factors associated with Overweight and Obesity in univariate and multivariate logistic regression among Urban Population

Characteristics	Overweight Vs Normal						Obesity Vs Normal					
	Unadjusted			Adjusted			Unadjusted			Adjusted		
	OR	95% CI	p-value	OR	95% CI	p-value	OR	95% CI	p-value	OR	95% CI	p-value
**Age**	1.02	1.01, 1.03	**<0.001**	1	0.99, 1.01	0.86	1.03	1.02, 1.04	**<0.001**	1.01	1.00, 1.02	0.18
**Sex**												
Women	Ref						Ref					
Men	1.02	0.78, 1.33	0.88				0.66	0.43, 1.02	0.06			
**Ecological Region**												
Mountain	Ref			Ref			Ref					
Hillly	2.49	1.30, 4.79	**0.01**	2.33	1.45, 3.75	**<0.001**	1.74	0.59, 5.13	0.32			
Terai	1.22	0.65, 2.26	0.53	1.57	0.96, 2.56	0.07	0.87	0.30, 2.51	0.79			
**Province**												
Koshi	Ref			Ref			Ref			Ref		
Madhesh	0.89	0.47, 1.66	0.7	0.96	0.52, 1.78	0.91	0.72	0.30, 1.71	0.45	0.73	0.31, 1.69	0.46
Bagmati	2.03	1.11, 3.70	**0.02**	1.82	1.18, 2.82	**0.007**	2.36	1.11, 5.04	**0.03**	2.36	1.07, 5.19	**0.033**
Gandaki	1.25	0.69, 2.28	0.46	1.1	0.70, 1.72	0.68	1.62	0.72, 3.63	0.24	1.46	0.61, 3.47	0.39
Lumbini	0.76	0.41, 1.39	0.36	0.86	0.43, 1.74	0.68	0.85	0.32, 2.26	0.74	0.78	0.27, 2.23	0.64
Karnali	0.42	0.23, 0.76	**<0.001**	0.36	0.22, 0.58	**<0.001**	0.4	0.13, 1.31	0.13	0.51	0.16, 1.69	0.27
Sudurpashchim	0.32	0.19, 0.55	**<0.001**	0.42	0.26, 0.66	**<0.001**	0.32	0.12, 0.84	**0.02**	0.4	0.14, 1.14	0.087
**Education**												
No formal education	Ref						Ref					
Primary	0.95	0.66, 1.36	0.78				1	0.61, 1.65	0.99			
Secondary(12yrs)	0.95	0.70, 1.28	0.74				0.69	0.39, 1.20	0.18			
University	1.46	0.84, 2.55	0.18				1.16	0.45, 2.99	0.76			
**Wealth Quintile**												
Lower Index	Ref						Ref					
Middle Index	0.91	0.64,1.30	0.60				0.93	0.69,1.24	0.61			
Upper Index	1.21	0.93,1.58	0.16				0.98	0.79,1.22	0.88			
**Ethnicity**												
Advantaged Janajatis	Ref			Ref			Ref			Ref		
Dalits	0.53	0.31, 0.89	**0.02**	0.71	0.44, 1.14	0.15	0.35	0.15, 0.79	**0.01**	0.49	0.20, 1.17	0.11
Disadvantaged Janajatis	0.44	0.29, 0.68	**<0.001**	0.52	0.36, 0.78	**0.001**	0.43	0.22, 0.85	**0.02**	0.45	0.22, 0.92	**0.028**
Religious Minorities	0.7	0.37, 1.34	0.28	1.07	0.54, 2.14	0.84	0.47	0.10, 2.18	0.33	0.85	0.18, 4.04	0.84

Characteristics	Overweight Vs Normal						Obesity Vs Normal					
	Unadjusted			Adjusted			Unadjusted			Adjusted		
	OR	95% CI	p-value	OR	95% CI	p-value	OR	95% CI	p-value	OR	95% CI	p-value
Upper caste	0.66	0.42, 1.05	0.08	0.85	0.57, 1.27	0.43	0.57	0.32, 1.03	0.06	0.75	0.40, 1.40	0.37
**Martial Status**												
Unmarried	Ref			Ref			Ref			Ref		
Ever Married	3.07	1.73, 5.44	**<0.001**	2.11	1.07, 4.13	**0.03**	3.41	1.03, 11.3	**0.05**	1	0.22, 4.50	>0.99
**Occupation**												
Unemployed	Ref			Ref			Ref			Ref		
Students	0.32	0.11, 0.96	**0.04**	0.78	0.31, 1.97	0.59	0.1	0.01, 0.66	**0.02**	0.18	0.02, 1.56	0.12
Home maker	0.96	0.42, 2.22	0.93	1.21	0.66, 2.19	0.53	1.34	0.40, 4.54	0.64	1.65	0.56, 4.84	0.36
Employed	1.15	0.48, 2.78	0.75	1.5	0.80, 2.81	0.2	1.92	0.55, 6.70	0.3	2.6	0.91, 7.42	0.073
Others	0.67	0.26, 1.73	0.41	0.9	0.42, 1.90	0.77	0.52	0.11, 2.45	0.41	0.91	0.22, 3.79	0.9
**Current Smoking**												
No	Ref						Ref					
Yes	0.9	0.61, 1.33	0.6				0.59	0.33, 1.07	0.08			
**Alcohol Consumption**												
No	Ref						Ref					
Yes	1.12	0.83, 1.50	0.45				0.78	0.48, 1.28	0.33			
**Sufficient Fruits and Vegetables**												
Insufficient	Ref						Ref					
Sufficient	1.19	0.76, 1.84	0.44				0.87	0.43, 1.75	0.69			
**Sufficient Physical Activity**												
Insufficient	Ref						Ref			Ref		
Sufficient	0.74	0.53, 1.03	0.08				0.57	0.36, 0.91	**0.02**	0.63	0.39, 1.02	0.061
**24 hours Salt Intake**												
No	Ref			Ref			Ref					
Yes	1.5	1.03, 2.18	**0.03**	1.39	0.86, 2.24	0.17	0.96	0.58, 1.59	0.88			
**Cholesterol**												
No	Ref			Ref			Ref			Ref		
Yes	1.01	1.00, 1.01	**<0.001**	1	1.00, 1.01	**0.01**	1.01	1.01, 1.02	**<0.001**	1.01	1.01, 1.02	**<0.001**

Table 3 depicts the factors associated with pre-hypertension and hypertension in multivariate logistic regression among urban population.

Age is found to be significantly associated with hypertension (AOR= 1.03,95%CI 1.02-1.04,p=0.001). In addition, male gender was

significantly associated with both pre-hypertension (AOR = 1.68, 95% CI = 1.19-2.36, P = 0.003) and hypertension (AOR = 2.23, 95% CI = 1.56-3.47, P = 0.001) with higher odds. Likewise, being in upper caste had the higher odds of prehypertension (AOR = 1.78, 95% CI = 1.13-2.79, P = 0.013). Furthermore, occupation wise, students had the lower odds of hypertension (AOR=0.25, 95%CI 0.09-0.68, p=0.007) while home makers (AOR = 2.15, 95% CI = 1.12-4.11, P = 0.021) and the “other” category (AOR = 3.14, 95% CI = 1.31-7.52, P = 0.011) had the higher odds of prehypertension as compared to the unemployed. As compared to normal weight individuals, underweight (AOR = 0.54, 95% CI = 0.34-0.87, P = 0.011) was significantly associated with pre-hypertension. Similarly, alcohol consumption (AOR = 1.58, 95% CI = 1.08-2.31, P = 0.019), cholesterol (AOR = 1.03, 95% CI = 1.02-1.04, P = 0.001), being overweight (AOR = 1.69, 95% CI = 1.19-2.39, P = 0.003), and being obese (AOR = 5.12, 95% CI = 2.95-8.87, P = 0.001) were significantly associated with hypertension.

Table 3: Factors associated with Pre-Hypertension and Hypertension in univariate and multivariate logistic regression among Urban Population

Characteristics	Pre-Hypertension Vs Normal						Hypertension Vs Normal					
	Unadjusted			Adjusted			Unadjusted			Adjusted		
	OR	95% CI	p-value	OR	95% CI	p-value	OR	95% CI	p-value	OR	95% CI	p-value
**Age**	1.03	1.02, 1.04	**<0.001**	1.01	1.00, 1.02	0.11	1.05	1.04, 1.06	**<0.001**	1.03	1.02, 1.04	**<0.001**
**Sex**												
Women	Ref			1			1			1		
Men	1.41	1.09, 1.84	**0.01**	1.68	1.19, 2.36	**0.003**	1.98	1.53, 2.57	**<0.001**	2.33	1.56, 3.47	**<0.001**
**Ecological Region**												
Mountain	1						1					
Hilly	1.33	0.70, 2.52	0.38				1.33	0.71, 2.46	0.37			
Terai	1.37	0.73, 2.58	0.32				1.04	0.58, 1.85	0.91			
**Province**												
Koshi	1						1					
Madhesh	1.1	0.66, 1.85	0.71				0.82	0.42, 1.57	0.54			
Bagmati	0.7	0.42, 1.17	0.17				0.93	0.48, 1.79	0.83			
Gandaki	1.16	0.75, 1.81	0.5				1.34	0.74, 2.40	0.33			
Lumbini	1.08	0.64, 1.81	0.77				1.25	0.68, 2.32	0.47			
Karnali	1.1	0.68, 1.77	0.69				0.84	0.43, 1.63	0.6			
Sudurpashchim	1.11	0.70, 1.74	0.66				0.88	0.45, 1.72	0.71			
**Education**												
No formal education	1						1			1		
Primary	1.14	0.79, 1.65	0.47				0.84	0.63, 1.13	0.24	1.14	0.80, 1.62	0.48
Secondary(12yrs)	0.84	0.61, 1.16	0.28				0.39	0.28, 0.54	**<0.001**	0.65	0.41, 1.03	0.065
University	0.52	0.24, 1.13	0.1				0.51	0.22, 1.20	0.12	0.71	0.22, 2.26	0.56
**Wealth Quintile**												
Lower Index	Ref						Ref					
Middle Index	1.14	0.84,1.56	0.39				0.97	0.67,1.41	0.88			
Upper Index	1.16	0.91,1.47	0.24				0.99	0.75,1.29	0.92			
**Wealth Quintile**												
**Ethnicity**												
Advantaged Janajatis	1			1			1					
Dalits	1.25	0.67, 2.33	0.49	1.23	0.67, 2.26	0.51	1.45	0.88, 2.39	0.15			
Disadvantaged categorys	1.2	0.75, 1.94	0.44	1.22	0.73, 2.01	0.45	0.75	0.47, 1.20	0.23			

Characteristics	Pre-Hypertension Vs Normal						Hypertension Vs Normal					
	Unadjusted			Adjusted			Unadjusted			Adjusted		
	OR	95% CI	p-value	OR	95% CI	p-value	OR	95% CI	p-value	OR	95% CI	p-value
Religious Minorities	1.65	0.96, 2.82	0.07	1.51	0.81, 2.83	0.19	0.89	0.48, 1.67	0.72			
Upper caste	1.56	1.00, 2.45	0.05	1.78	1.13, 2.79	**0.013**	0.88	0.57, 1.37	0.57			
**Marital Status**												
Unmarried	1			1			1			1		
Ever Married	1.75	1.21, 2.54	**<0.001**	1.27	0.63, 2.54	0.5	3.76	2.29, 6.18	**<0.001**	0.73	0.39, 1.40	0.34
**Occupation**												
Unemployed	1			1			1			1		
Students	1.06	0.51, 2.19	0.87	1.61	0.61, 4.21	0.33	0.1	0.04, 0.25	**<0.001**	0.25	0.09, 0.68	**0.007**
Home maker	1.81	1.00, 3.25	**0.05**	2.15	1.12, 4.11	**0.021**	1.32	0.73, 2.38	0.35	1.62	0.90, 2.90	0.1
Employed	1.86	0.99, 3.46	**0.05**	1.68	0.85, 3.32	0.14	2.3	1.28, 4.12	**0.01**	1.82	0.96, 3.44	0.066
Others	2.9	1.33, 6.32	**0.01**	3.14	1.31, 7.52	**0.011**	1.69	0.79, 3.60	0.18	2.07	0.92, 4.66	0.079
**Current Smoking**												
No	1						1			1		
Yes	0.59	0.33, 1.07	0.08				1.76	1.28, 2.42	**<0.001**	0.86	0.58, 1.27	0.45
**Alcohol Consumption**												
No	1						1			1		
Yes	1.29	0.93, 1.79	0.13				2.34	1.82, 3.02	**<0.001**	1.58	1.08, 2.31	**0.019**
**Sufficient Fruits and Vegetables**												
Insufficient	1						1					
Sufficient	1.35	0.92, 1.97	0.12				1.06	0.73, 1.54	0.76			
**Sufficient Physical Activity**												
Insufficient	1						1					
Sufficient	1.06	0.77, 1.46	0.71				1.31	0.93, 1.86	0.13			
**24 hours Salt Intake**												
No	1						1					
Yes	0.98	0.67, 1.43	0.92				0.91	0.65, 1.28	0.58			
**Cholesterol**	1.03	1.02, 1.04	**<0.001**	1.01	1.00, 1.02	0.11	1.05	1.04, 1.06	**<0.001**	1.03	1.02, 1.04	**<0.001**
**Obesity**												
Normal	1			1			1			1		
Obesity	2.15	1.27, 3.64	**<0.001**	1.65	0.94, 2.90	0.083	5.85	3.71, 9.24	**<0.001**	5.12	2.95, 8.87	**<0.001**
Overweight	1.23	0.85, 1.79	0.27	1.01	0.69, 1.48	0.95	1.88	1.40, 2.51	**<0.001**	1.69	1.19, 2.39	**0.003**
Underweight	0.55	0.35, 0.86	**0.01**	0.54	0.34, 0.87	**0.011**	0.61	0.37, 1.01	0.06	0.6	0.36, 1.00	0.05

Table 4 shows the factors associated with pre-diabetics and diabetics among urban population. Ecological region and obesity showed significant associations with pre-diabetes. Compared to the mountain region, urban population residing in the hilly region had substantially higher odds of pre-diabetes (AOR = 3.59, 95% CI = 1.6-8.08, p = 0.002), and those in the Terai region had even higher odds (AOR = 5.07, 95% CI = 2.44-10.5, p < 0.001) of pre-diabetes. Being obese (AOR=1.82, 95% CI=1.05-3.16, P=0.033) and high cholesterol level (AOR=1.01,95%CI=1.01-1.01 p=0.001) was also significantly associated with being pre-diabetic. Age, ecological region, province, education, ethnicity, cholesterol and obesity were found significantly associated with diabetes. For each one-year increase in age, the odds of having diabetes increased by approximately 2% (AOR = 1.02, 95% CI = 1.01-1.04, p = 0.011). The ecological region remained a strong predictor of diabetes. Urban population residing in the Hilly region (AOR = 6.44, 95% CI = 3.31-11.10, p = 0.002) and Terai region (AOR = 5.96, 95% CI = 3.12-19.86, p = 0.001) had remarkably higher odds of diabetes compared to those in the Mountain region. Urban population living in Madhesh province had higher odds of being diabetic (AOR = 3.1, 95% CI = 1.23-7.83, p = 0.017) compared to the Koshi province. University level education had higher odds of being diabetic (AOR = 3.98, 95% CI = 1.39-11.44, p = 0.011 compared to no formal education. Ethnicity also showed significant associations with diabetes. Religious minorities had the highest odds of diabetes (AOR = 3.76, 95% CI = 1.11-12.73, p = 0.034) compared to advantaged Janajatis. Having high cholesterol (AOR=1.01, 95% CI= 1.01-1.02, P=0.001) and obesity (AOR=2.41, 95% CI= 1.06-5.48, P=0.037) had higher odds of being diabetic compared to people wth normal cholesterol levels and normal weight, respectively.

Table 4: Factors associated with Pre-Diabetics and Diabetics in univariate and multivariate logistic regression among Urban Population

Characteristics	Pre-Diabetics Vs Normal						Diabetics Vs Normal					
	Unadjusted			Adjusted			Unadjusted			Adjusted		
	OR	95% CI	p-value	OR	95% CI	p-value	OR	95% CI	p-value	OR	95% CI	p-value
**Age**	1.02	1.01, 1.03	**<0.001**	1.01	1.00, 1.02	0.11	1.04	1.02, 1.05	**<0.001**	1.02	1.01, 1.04	**0.011**
**Sex**												
Women	1						1					
Men	1.15	0.88, 1.51	0.29				1.12	0.75, 1.68	0.58			
**Ecological Region**												
Mountain	1			1			1			1		
Hilly	2.99	1.21, 7.39	**0.02**	3.59	1.6, 8.08	**0.002**	3.88	0.66, 22.9	0.13	6.44	3.31, 11.10	**0.002**
Terai	4.37	1.90, 10.09	**<0.001**	5.07	2.44, 10.5	**<0.001**	5.84	1.43, 8.96	**<0.001**	5.96	3.12, 19.86	**<0.001**
**Province**												
Koshi	1						1			1		
Madhesh	1.1	0.66, 1.85	0.71				3.52	1.51, 8.20	**<0.001**	3.1	1.23, 7.83	**0.017**
Bagmati	0.7	0.42, 1.17	0.17				0.83	0.34, 2.01	0.67	1.15	0.51, 2.56	0.74
Gandaki	1.16	0.75, 1.81	0.5				0.78	0.30, 1.99	0.59	1.03	0.43, 2.49	0.94
Lumbini	1.08	0.64, 1.81	0.77				1.85	0.77, 4.45	0.17	1.26	0.58, 2.70	0.56
Karnali	1.1	0.68, 1.77	0.69				0.1	0.03, 0.29	**<0.001**	0.28	0.08, 1.01	0.052
Sudurpashchim	1.11	0.70, 1.74	0.66				1	0.28, 3.64	1	1.15	0.23, 5.70	0.86
**Education**												
No formal education	1			1			1			1		
Primary	1.02	0.71, 1.49	0.9	1.24	0.80, 1.92	0.34	0.79	0.46, 1.33	0.36	1.47	0.76, 2.84	0.25
Secondary(12yrs)	0.64	0.45, 0.93	**0.02**	0.84	0.55, 1.27	0.4	0.49	0.30, 0.81	**0.01**	1.17	0.61, 2.23	0.64
University	0.43	0.25, 0.75	**<0.001**	0.61	0.33, 1.13	0.11	0.91	0.35, 2.37	0.85	3.98	1.39, 11.44	**0.011**
**Wealth Quintile**												
Lower Index	Ref						Ref					
Middle Index	1.28	0.92,1.78	0.14				0.97	0.67,1.41	0.88			
Upper Index	1.26	0.95,1.66	0.10				0.99	0.75,1.29	0.92			
**Ethnicity**												
Advantaged Janajatis	1						1			1		
Dalits	0.91	0.45, 1.82	0.78				1.16	0.43, 3.14	0.76	1.18	0.42, 3.28	0.75
Disadvantaged categories	1.13	0.61, 2.08	0.7				2.15	1.14, 4.08	**0.02**	1.44	0.75, 2.77	0.28
Religious Minorities	1.17	0.52, 2.63	0.7				6.22	2.39, 16.1	**<0.001**	3.76	1.11, 12.73	**0.034**
Upper casts	0.8	0.44, 1.47	0.47				1.32	0.52, 3.35	0.56	1.18	0.54, 2.57	0.68
**Marital Status**												
Unmarried	1			1			1			1		
Ever Married	1.62	1.11, 2.34	**0.01**	1	0.66, 1.52	0.98	3.8	1.54, 9.38	**<0.001**	1.31	0.23, 7.54	0.76
**Occupation**												

Characteristics	Pre-Diabetics Vs Normal						Diabetics Vs Normal					
	Unadjusted			Adjusted			Unadjusted			Adjusted		
	OR	95% CI	p-value	OR	95% CI	p-value	OR	95% CI	p-value	OR	95% CI	p-value
Unemployed	1						1			1		
Students	0.65	0.27, 1.56	0.33				0.52	0.14, 1.92	0.33	0.86	0.10, 7.13	0.89
Home maker	1.46	0.71, 3.02	0.31				2.11	0.73, 6.09	0.17	1.23	0.41, 3.67	0.71
Employed	1.7	0.82, 3.52	0.15				1.38	0.48, 3.97	0.54	0.81	0.28, 2.34	0.7
Others	2.01	0.93, 4.34	0.07				3.1	1.03, 9.31	0.04	1.76	0.55, 5.67	0.34
**Current Smoking**												
No	1						1					
Yes	1.19	0.86, 1.65	0.3				1.54	0.86, 2.75	0.15			
**Alcohol Consumption**												
No	1						1					
Yes	1.03	0.77, 1.38	0.85				0.92	0.55, 1.54	0.75			
**Sufficient Fruits and Vegetables**												
Insufficient	1			1			1					
Sufficient	1.44	0.99, 2.09	0.05	1.3	0.88, 1.92	0.19	1.53	0.76, 3.05	0.23			
**Sufficient Physical Activity**												
Insufficient	1						1					
Sufficient	0.85	0.60, 1.23	0.39				0.98	0.59, 1.63	0.94			
**24 hours Salt Intake**												
No	1						1					
Yes	1.39	0.92, 2.10	0.11				0.88	0.51, 1.54	0.66			
**Cholesterol**	1.01	1.01, 1.02	**<0.001**	1.01	1.01, 1.01	**<0.001**	1.02	1.01, 1.02	**<0.001**	1.01	1.01, 1.02	**<0.001**
**Obesity**												
Normal	1			1			1			1		
Obesity	2.21	1.32, 3.68	**<0.001**	1.82	1.05, 3.16	**0.033**	3.3	1.63, 6.70	**<0.001**	2.41	1.06, 5.48	**0.037**
Overweight	1.22	0.88, 1.68	0.23	1.1	0.81, 1.49	0.53	1.32	0.68, 2.58	0.41	1.06	0.46, 2.45	0.9
Underweight	1.01	0.67, 1.53	0.95	0.96	0.64, 1.45	0.86	0.57	0.29, 1.12	0.1	0.59	0.28, 1.24	0.16
**Hypertension**												
Normal	1			1			1			1		
Pre-Hypertension	1.32	0.95, 1.84	0.1	1.12	0.79, 1.58	0.53	1.98	1.01, 3.90	**0.05**	1.44	0.74, 2.84	0.28
Hypertension	1.49	1.08, 2.06	**0.02**	1.06	0.74, 1.52	0.74	2.93	1.59, 5.38	**<0.001**	1.84	0.99, 3.41	0.054

Discussion

Overall, our analysis highlights the association between socio-economic determinants, NCD risk behaviors and NCD within the urban population. Our analysis revealed notable disparities in education and occupation between the urban population, showing the socio-economic factors contributing to these differences. As revealed in the analysis, those with higher levels of education and employment are more prevalent in the middle and rich quintiles, whereas those with no formal education and homemakers are more prevalent in the poor quintile. Most urban population live in slums that are unregulated, have congested conditions, are overcrowded, are positioned near open sewers, and restricted to geographically dangerous areas such as hillsides, riverbanks, and water basins subject to flooding [18]. This has contributed to a growing gap between rich and poor in terms of adequate urban housing, employment opportunities, transportation, levels of education, and access to affordable health services of decent quality [19]. While our study did not find a significant association between NCDs and wealth quintile, studies had identified NCD risk factors and their relationship with socioeconomic status. Many studies had highlighted that health outcomes such as diabetes and hypertension are more prevalent among the wealthiest population, while risk factors are increasing among the urban poor. The absence of a clear association implies that NCD risk factors may pose a similar risk to individuals across varying wealth levels in urban areas, including the urban poor, urban middle and urban rich. This finding underscores the potential vulnerability of all urban segments of NCDs and emphasizes the need for targeted interventions and policies across diverse urban populations.

Age emerged as a significant predictor, with a consistent increase in the odds of hypertension and diabetes with advancing age. Studies identified that the burden of hypertension and diabetes typically begins to manifest in the adult population, when they reach adult age groups and these

conditions are often considered to be age-related, meaning their prevalence increases with age [20,21].

Sex-based differences were evident, with men exhibiting higher odds of pre-hypertension and hypertension compared to women. Epidemiological data consistently demonstrate a higher prevalence of hypertension in males compared to females across various age groups [8,20]. This discrepancy may be attributed to hormonal differences, as well as lifestyle and behavioral factors more prevalent in males, such as higher rates of tobacco and alcohol consumption [22,23]. Additionally, genetic predispositions and variations in physiological responses to stressors may contribute to this gender disparity [22,23]. However, contradictory findings were observed in a recent study, where female participants from India were more likely to be hypertensive than males, in comparison to women from Bangladesh and Nepal [24].

Likewise, urban populations residing in the Hilly region and Terai region had substantially higher odds of pre-diabetes and diabetes as compared to those residing in mountain region. Additionally, urban populations residing in the Madhesh province had higher odds of being diabetic. This pattern aligns with previous research indicating lower diabetes rates in high-altitude areas of mountain [25,26]. The lower oxygen levels in mountainous regions may influence glucose metabolism, potentially reducing diabetes risk. Furthermore, the mountainous terrain may encourage physical activity and a traditional dietary pattern, which are protective against diabetes [25,26]. In contrast, the Terai region's flatter topography and greater accessibility to modern amenities may lead to a higher prevalence of sedentary lifestyles and less healthy dietary practices, thereby elevating diabetes prevalence in that region [8,17].

Urban population in Karnali and Sudurpashchim provinces had lower odds of overweight compared to those in Koshi province, while urban population in Bagmati province had higher odds

of overweight and obesity compared to Koshi province. Factors contributing to these variations could include differences in lifestyle, access to healthcare services, and socio-economic conditions [8,10,27]. The higher odds of being overweight in Bagmati province could be attributed to factors such as concentration of urbanization by provinces (73% of population were urbanized), lifestyle changes, and potentially greater access to calorie-dense foods. The lower odds of being overweight and diabetic in Karnali and Sudurpashchim provinces may be due to less urbanization (25% of Urban Population in Karnali and 34% of Urban population in Sudurpashchim) and low socio-economic conditions [6,8,27].

The relationship between education level and diabetes is a noteworthy observation in our study. Those with university level education had lower odds of being diabetic compared to those with no formal education. Higher educational attainment is associated with a lower risk of both conditions. Urban population with higher education tends to have greater health literacy and awareness, leading to better self-care practices and healthier lifestyles [28,29]. They are also more likely to have access to resources for preventive healthcare and engage in regular check-ups. Moreover, education can empower individuals to make informed decisions about their diet, exercise, and overall well-being, reducing their susceptibility to diabetes [28-30]. These findings underscore the importance of educational interventions in public health strategies aimed at reducing the burden of these chronic conditions.

Marital Status was associated with being overweight. Marriage often brings about lifestyle changes, including shared meals and reduced time for individual exercise, which can contribute to weight gain [31]. Moreover, couples may engage in social activities that involve eating out or preparing larger portion sizes at home [32]. While the support and companionship of marriage offer numerous benefits, understanding the potential impact on weight and promoting healthy

behaviors within marital relationships is important for public health efforts aimed at reducing overweight and obesity [31-32].

The association between being a homemaker and prehypertension, as well as employment and hypertension, highlights intriguing dynamics in our study. Homemakers comprises of women who may be unemployed due to household duties, household management, childcare responsibilities, it also encompasses those who have chosen to prioritize home duties over formal employment. Homemakers may experience varying levels of financial stress and other stressors related to household responsibilities, childhood responsibilities, potentially contributing to hypertension. For employed population, the demands and pressures of employment may lead to higher stress levels, potentially elevating the risk of hypertension. Additionally, employment may influence lifestyle factors such as sedentary behavior and dietary habits, which can further contribute to hypertension risk [33,34].

The association between cholesterol and obesity, hypertension, and diabetes underscores the complex interplay of these risk factors in our study. Elevated cholesterol often coexists with these conditions due to shared underlying mechanisms. Obesity can lead to dyslipidemia and impaired lipid metabolism, resulting in elevated cholesterol levels [35]. Similarly, hypertension and diabetes can disrupt lipid profiles through metabolic pathways and oxidative stress. Additionally, these conditions can collectively contribute to atherosclerosis, further elevating cholesterol levels [36].

Obesity was associated with hypertension, prediabetes and diabetes. Extensive studies reveal a strong association between obesity, hypertension, and diabetes [37-40]. Obesity, particularly central adiposity, significantly raises the risk of developing hypertension, with adipose tissue secreting substances impacting blood pressure regulation [37,38]. Additionally, obesity is a major contributor to insulin resistance, linking it closely to the onset of type 2 diabetes [39,40]. The

coexistence of obesity, hypertension, and diabetes forms a cluster known as metabolic syndrome, heightening cardiovascular risks [38,39]. Lifestyle interventions, including weight management through balanced nutrition and physical activity, emerge as pivotal strategies for preventing and managing this intricate interplay of conditions, underlining the importance of holistic health approaches in mitigating these public health challenges.

Conclusion

In conclusion, our analysis underscores the intricate interplay between socio-economic determinants, NCD risk behaviors, and NCDs within urban populations. Factors such as education, occupation, age, gender, ecological region, and province contribute significantly to the prevalence of conditions like hypertension, diabetes, and overweight among the urban population. The disparities observed in different regions suggest the influence of diverse lifestyles, socio-economic conditions, and access to healthcare services. Additionally, the study highlights the role of education in reducing the risk of diabetes and hypertension, emphasizing the need for educational interventions in public health strategies. Understanding these dynamics is crucial for developing targeted interventions to address the complex web of factors contributing to the burden of NCDs among urban populations. Public health efforts should aim to create comprehensive strategies that address socio-economic disparities, promote healthy lifestyles, and enhance health literacy to effectively combat the rising prevalence of NCDs.

References

1. WHO. Noncommunicable diseases progress monitor 2022 [Internet]. 2022 [cited 2023 Sep 14]. Available from:

https://www.google.com/search?q=Noncommunicable+diseases+progress+monitor+2022.+

2. WHO. WHO. 2022 [cited 2023 Sep 14]. Noncommunicable diseases fact sheets. Available from: https://www.who.int/news-room/fact-sheets/detail/noncommunicable-diseases
3. Allen L, Williams J, Townsend N, Mikkelsen B, Roberts N, Foster C, et al. Socioeconomic status and non-communicable disease behavioural risk factors in low-income and lower-middle-income countries: a systematic review. Lancet Glob Health. 2017;5(3).
4. WHO. The global health observatory. Non-communicable disease risk factors. 2024 [cited 2024 May 11]. Available from: https://www.who.int/data/gho/data/themes/topics/topic-details/GHO/ncd-risk-factors
5. Gama e Colombo D. Closing the gap in a generation: health equity through action on the social determinants of health. Final report of the Commission on Social Determinants of Health. REV DIREITO SANIT. 2010;10(3).
6. Marmot M, Bell R. Social determinants and non-communicable diseases: Time for integrated action. BMJ (Online). 2019;364.
7. Solar, O., & Irwin, A. A conceptual framework for action on the social determinants of health. WHO Document Production Services. Discussion Paper Series. 2010;
8. Bista B, Dhimal M, Bhattarai S, Neupane T, Xu YY, Pandey AR, et al. Prevalence of non-communicable diseases risk factors and their determinants: Results from STEPS survey 2019, Nepal. Vol. 16, PLoS one. 2021.

9. Rosenberg, Paul, Megumi Kano, Isobel Ludford, Amit Prasad, and Hilary Thomson. Global report on urban health: equitable, healthier cities for sustainable development. World Health Organization, 2016.

10. World Health Organization (WHO). Urban Health Initiative. 2024. [cited 2024 Feb 18] https://www.who.int/initiatives/urban-health-initiative/pilot-projects/kathmandu. 2020.

11. Adhikari, A. P., Dahal, G. P., Mahat, I., Regmi, B., & Subedi, K. Sustainable Livelihood Systems in Nepal Principles, Practices and Prospects 14 Social Determinants of Health in Nepal: A Neglected Paradigm [Internet]. Available from: https://www.researchgate.net/publication/289540976

12. De Snyder VNS, Friel S, Fotso JC, Khadr Z, Meresman S, Monge P, et al. Social conditions and urban health inequities: Realities, challenges and opportunities to transform the urban landscape through research and action. J Urban Health. 2011;88(6).

13. Kuddus MA, Tynan E, McBryde E. Urbanization: A problem for the rich and the poor? Public Health Rev. 2020;41(1).

14. Friel S, Akerman M, Hancock T, Kumaresan J, Marmot M, Melin T, et al. Addressing the social and environmental determinants of urban health equity: Evidence for action and a research agenda. J Urban Health. 2011;88(5).

15. Central Bureau of Statistics. Preliminary Report of National Population. 2021. [cited 2023 19 December] Available from https://censusnepal.cbs.gov.np/Home/Details?tpid=5&dcid=3479c092-7749-4ba6-9369-45486cd67f30&tfsid=17

16. Dhimal M, Bista B, Bhattarai S, Dixit LP, Hyder MKA, Agrawal N, et al. Report of Noncommunicable Disease Risk Factors: STEPS Survey Nepal 2019. Kathmandu: Nepal Health Research Council, 2020.

17. Kuddus MA, Tynan E, McBryde E. Urbanization: A problem for the rich and the poor? Public Health Rev. 2020;41(1).

18. Friel S, Akerman M, Hancock T, Kumaresan J, Marmot M, Melin T, et al. Addressing the social and environmental determinants of urban health equity: Evidence for action and a research agenda. J Urban Health. 2011;88(5).

19. Fattahi N, Azadnajafabad S, Mohammadi E, Aminorroaya A, Rezaei S, Ghasemi E, et al. Geographical, gender and age inequalities in non-communicable diseases both at national and provincial levels in Iran. J Diabetes Mellit Metab Syndr. 2021 Feb:1-7.

20. Sapkota BP, Baral KP, Rehfuess EA, Parhofer KG, Berger U. Effects of age on non-communicable disease risk factors among Nepalese adults. PLoS one. 2023 Jun 2;18(6):e0281028.

21. Oli N, Vaidya A, Thapa G. Behavioural risk factors of noncommunicable diseases among Nepalese urban poor: a descriptive study from a slum area of Kathmandu. Epidemiology research international. 2013 Nov 4;2013.

22. Varì R, Scazzocchio B, D’Amore A, Giovannini C, Gessani S, Masella R. Gender-related differences in lifestyle may affect health status. ANN I SUPER SANITA. 2016 Jun 28;52(2):158-66.

23. Sharma SK, Vishwakarma D, Puri P. Gender disparities in the burden of non-communicable diseases in India: evidence from the cross-sectional study. Clin Epidemiol Glob Health. 2020 Jun 1;8(2):544-9.

24. Mohajeri S, Perkins BA, Brubaker PL, Riddell MC. Diabetes, trekking and high altitude: recognizing and preparing for the risks. Diabetic Medicine. 2015 Nov;32(11):1425-37.

25. Burtscher M. Effects of living at higher altitudes on mortality: a narrative review. Aging and disease. 2014 Aug;5(4):274.

26. Dhungana RR, Karki KB, Bista B, Pandey AR, Dhimal M, Maskey MK. Prevalence, pattern and determinants of chronic disease multimorbidity in Nepal: secondary analysis of a national survey. BMJ open. 2021 Jul 1;11(7):e047665.

27. Niessen LW, Mohan D, Akuoku JK, Mirelman AJ, Ahmed S, Koehlmoos TP, et al. Tackling socioeconomic inequalities and non-communicable diseases in low-income and middle-income countries under the Sustainable Development agenda. Lancet. 2018 May 19;391(10134):2036-46.

28. Heine M, Lategan F, Erasmus M, Lombaard CM, Mc Carthy N, Olivier J, et al. Health education interventions to promote health literacy in adults with selected non-communicable diseases living in low-to-middle income countries: A systematic review and meta-analysis. J. Eval. Clin. Pract.. 2021 Dec;27(6):1417-28.

29. Hosseinpoor AR, Bergen N, Mendis S, Harper S, Verdes E, Kunst A, et al. Socioeconomic inequality in the prevalence of noncommunicable diseases in low-and middle-income countries: results from the World Health Survey. BMC public health. 2012 Dec;12(1):1-3.

30. Rana K, Ghimire P, Chimoriya R, Chimoriya R. Trends in the prevalence of overweight and obesity and associated socioeconomic and household environmental factors among women in Nepal: findings from the Nepal demographic and health surveys. Obesities. 2021 Sep 2;1(2):113-35.

31. Shariful Islam M, Ola O, Alaboson J, Dadzie J, Hasan M, Islam N, et al. Trends and socioeconomic factors associated with overweight/obesity among three reproductive age groups of women in Nepal. Lifestyle Medicine. 2022 Jan;3(1):e51.

32. Shrestha S, Devkota R. Prevalence of hypertension and its associated risk factors in a sub-urban area of central Nepal. Int J Community Med Public Health. 2016 Sep;3(9):2477-86.

33. Tipayamongkholgul M, Kongtip P, Woskie S. Association between occupations and selected noncommunicable diseases: A matched case-control among Thai informal workers. J. Occup. Health. 2021;63(1):e12249.

34. Nakamura Y, Okamura T, Tamaki S, Kadowaki T, Hayakawa T, Kita Y, et al. Egg consumption, serum cholesterol, and cause-specific and all-cause mortality: the National Integrated Project for Prospective Observation of Non-communicable Disease and Its Trends in the Aged, 1980 (NIPPON DATA80). Am J Clin Nutr. 2004 Jul 1;80(1):58-63.

35. Thankappan KR, Shah B, Mathur P, Sarma PS, Srinivas G, Mini GK, et al. Risk factor profile for chronic non-communicable diseases: results of a community-based study in Kerala, India. Indian J. Med. Res. 2010 Jan 1;131(1):53-63.

36. Macmohan S, Cutler J, Brittain E, Higgins M. Obesity and hypertension: epidemiological and clinical issues. Eur Heart J. 1987 May 1;8(suppl_B):57-70.

37. Sharma SK, Ghimire A, Radhakrishnan J, Thapa L, Shrestha NR, Paudel N, et al. Prevalence of hypertension, obesity, diabetes, and metabolic syndrome in Nepal. Int. J. Hypertens. 2011 Apr 19;2011.

38. Hossain P, Kawar B, El Nahas M. Obesity and diabetes in the developing world—a growing challenge. N Engl J Med . 2007 Jan 18;356(3):213-5.

39. Colosia AD, Palencia R, Khan S. Prevalence of hypertension and obesity in patients with type 2 diabetes mellitus in observational studies: a systematic literature review. Diabetes Metab. Syndr. Obes.: Targets Ther. 2013 Sep 17:327-38.

Click here to access/download
Supporting Information
Title Page.docx
