# Supplementary material for: Social determinants and risk factors associated with non-communicable diseases among urban population in Nepal: A comparative study of poor, middle and rich wealth categories of urban population using STEPS survey
Source: PLoS One. 2025 May 14;20(5):e0307622. doi: 10.1371/journal.pone.0307622 (PMC12077703; doi:10.1371/journal.pone.0307622)
Supplement: Attachment — Submitted filename: 2_Editors Report@@PONE-D-24-27355.pdf [file pone.0307622.s005.pdf]

Editors' Feedback:

Version: 1 **Date:** Aug 25, 2024

MS: PONE-D-24-27355

Title: *Social determinants and risk factors linked to non-communicable diseases among urban populations in Nepal: A comparative analysis across poor, middle, and affluent wealth groups using the STEPS survey*.

General Feedback:

In this study, the authors examined the prevalence and contributing factors of non-communicable diseases (NCDs) within various wealth groups in Nepal.

This research introduces new data regarding the prevalence and relationship between NCDs and contextual factors, highlighting a crucial area of exploration. Nevertheless, the authors should address concerns regarding potential chance findings due to the extensive number of associations analyzed. To mitigate this, authors may consider applying a Bonferroni correction.

Despite these issues, the study offers valuable data and insights. I therefore suggest resubmitting the paper with revisions to address these points.

Typographical Errors

Abstract:

- Urban population Hilly region → Urban population from the Hilly region

Introduction:

- Typographical errors:
  - Socio-economic factors are associated with behavioral risk factors ~~for~~ and mortality from NCDs.
- The phrases "Rapid urbanization, connection with vulnerable populations, and elevated NCDs risk" are unclear and challenging for readers. Please clarify what the authors intend to convey, particularly why the detection rate is increasing.
- The statement "*Urban residents face material constraints, limiting access to nutritious food, quality healthcare, and the ability to maintain healthy lifestyles*" requires further elaboration. How do these constraints manifest?
- The authors should expand their literature review to encompass the latest epidemiological knowledge, particularly in relation to the social determinants of NCDs, both in Nepal and internationally. This will help identify gaps in knowledge and strengthen the rationale for the study, as reviewers have also suggested.

Methodology:

- In the Site selection section, numerous data points lack citations.
- Clarify the term "weighted" in the context of multivariate logistic regression models.
- Include the NHRC Ethical Approval Number.

Results:

- Given the extensive number of associations examined, there is a high likelihood of chance findings. Therefore, applying Bonferroni correction would be advisable.

Discussion:

- The interpretation that "*This finding underscores the potential vulnerability of all urban segments to NCDs and emphasizes the need for targeted interventions and policies across diverse urban populations*" does not align with the results and previous statements. Please revise.
- The statement "*This discrepancy may be attributed to hormonal differences, as well as lifestyle and behavioral factors more prevalent in males, such as higher rates of tobacco and alcohol consumption*" should be directly linked to the study data on alcohol and smoking, rather than relying on citations.
- Prioritize the application of Bonferroni correction, as only a few associations will likely remain significant, which should be the focus of the discussion.

Despite these concerns, the comprehensive analysis provides valuable new insights. I recommend resubmission with revisions to address these points including that of 3 expert reviewers.
