# Supplementary material for: Social determinants and risk factors associated with non-communicable diseases among urban population in Nepal: A comparative study of poor, middle and rich wealth categories of urban population using STEPS survey
Source: PLoS One. 2025 May 14;20(5):e0307622. doi: 10.1371/journal.pone.0307622 (PMC12077703; doi:10.1371/journal.pone.0307622)
Supplement: Attachment — Submitted filename: PONE-D-24-27355_Reviewer-comments.docx [file pone.0307622.s003.docx]

The manuscript “PONE-D-24-27355” entitled “Social determinants and risk factors associated with non-communicable diseases among urban population in Nepal: A comparative study of poor, middle and rich wealth categories of urban population using STEP survey” in itself is well written and represents a comprehensive study in relation to the risk factors for non-communicable diseases (NCDs). While scanning the manuscript thoroughly, it is understood that authors have clearly mentioned the methodological design with multistage stratified technique. One of the crucial parts of this paper is noticed that it has incorporated almost all socio-demographical parameters which might contribute as risk factors for NCDs. However, some suggestions are provided below which would enable further strength of the manuscript and imply its importance in public health domain. This paper can be considered provided the below mentioned aspects are satisfactorily addressed. In a nutshell, a minor revision is required.

Suggestions/Comments:

1. This manuscript is silent regarding the hypothesis of the study which is deemed pertinent in such kind of public health related analytical study. It would be fine to mention the hypothesis briefly in the last paragraph of the “Introduction” section. Please consider it in the revised MS.
2. As mentioned in Table 1, what does the term “average day” indicate? Please specify.
3. The variable “24 hours salt consumption” seems to be poorly defined (Table 1). Please redefine it.
4. The term “Terai” used in the MS should be mentioned as “Tarai” as it is a typical Nepali naming and should look like an original one.
5. In Methods section or elsewhere, a country map of Nepal indicating the proportion of study participants representing the provincial location and ecological belt would be worth incorporating. If possible, the dominant associated risk factors for NCDs be mentioned with specific notation (i.e., with different color code or same color with decreasing intensity) in the specific ecological and political zone of the country. This would provide readers an overall picture of the entire study.
6. Minor typo errors need to be corrected throughout the MS.
