# Supplementary material for: Social determinants and risk factors associated with non-communicable diseases among urban population in Nepal: A comparative study of poor, middle and rich wealth categories of urban population using STEPS survey
Source: PLoS One. 2025 May 14;20(5):e0307622. doi: 10.1371/journal.pone.0307622 (PMC12077703; doi:10.1371/journal.pone.0307622)
Supplement: Attachment — Submitted filename: Comments to authors_Manuscript PONE-D-24-27355.docx [file pone.0307622.s004.docx]

**Comments to the authors:**

Thank you for the opportunity to review this paper. This manuscript specifically compared the social determinants, NCD risks and prevalence among different wealth categories and determined the factors associated with different NCDs among urban population in Nepal. Given the limited epidemiological evidence on the risk factors and determinants of NCDs in low- and middle-income countries, including Nepal, this study had tried to address a growing concern of the impact of increasing urbanization and how socioeconomic status affects NCD risk and prevalence within urban settings using the recent data from a 2019 STEPS survey from Nepal. However, I have several comments and suggestions which might be helpful to improve the manuscript.

**Abstract:**

Methods:

- Study design is not mentioned
- You can specify the age group of the participants.

Results: You may include some important result data from table 1

Conclusion: Recommendations are quite general and are not specific based on the study findings. I would suggest making them more specific that align with the study findings.

Minor points:

- In line 32, you can add acronym for STEPwise approach to NCD risk factor surveillance
- In line 41, spell out AOR when it appeared for the first time in the abstract also.

**Introduction:**

- Please cite more previous studies from Nepal to explain why this study is important among urban population in Nepal.
- In the last paragraph, the authors mentioned “this study aims to inform effective strategies that mitigate the burden of NCDs and enhance the overall well-being of the urban population in Nepal.” Was this also the study objective and how was this done from this study?
- Line 74: grammar, ‘for’ after behavioral risk factors may be removed.
- Line 94-95: add citation to the sentence ‘The dynamics of urban health in Nepal is associated with a complex and interlinked range of wider determinants’
- Lines 99-100: add citation to the line “Furthermore, increasing rates….complexity to public health challenges”.
- Lines 102-104: add citation to the line “the co-influence of socio-economic factors…..social determinants and health outcomes.

**Methods:**

Study design and sampling size:

- Please provide reference of the STEPS survey.
- The section title mentions "sampling size", but no specific information on sample size calculation is provided. Sampling size may not be the right word, instead sampling technique might be better.
- Under ‘Study design and sampling size’, information about data collection and survey instrument is also mentioned. You may create a separate section for this, and you can explain more about survey questions.

Study setting:

- It might be better to include ‘study setting’ first, followed by study design and sampling. Study setting can help establish the context of the study design and sampling methods.
- While the description of Nepal's geography is informative in study setting, the description is a bit long compared to other sections. It would be better to make it shorter and link it with the study design and sampling in next paragraph.
- There is an error in the urbanization statistics. Line 144 "66% of the population living in urban areas in 2021 compared to just 23% in 2021" uses the same year twice, which does not make sense. The years should be different for this comparison.
- Please provide a citation for the urbanization and population distribution statistics provided in the study setting, particularly if they are not from the STEPS survey data.

Participant:

- First sentence has already been mentioned in the section ‘study design and sampling size section’ and has been repeated here.
- Authors have mentioned that they included 3,460 individuals from urban areas in the analysis. It is not clear how 3,460 participants for this study were selected from the total population (6,475). The definition of urban area is not clear in the study and should be clearly defined.
- This section also includes information about sampling technique.

Variable definitions:

- How was salt consumption measured in the STEPS survey and how was this defined in this study? Given that salt is necessary nutrient for our body in smaller amount and over consumption could be harmful, did the participants respond as ‘yes’ or ‘no’ for salt consumption in the past 24 hours as shown in table 1?
- Please check the definition of pre-hypertension, hypertension, and pre-diabetes.

Statistical analysis:

- Three categories were created from 5 wealth quintiles creating unevenly distributed categories. What was the reason for authors to merge categories? Did you try to analyze data using five original categories (5 quintiles)?
- In the analysis, authors mentioned that they included variables one at a time in the univariate analysis and then including significant variables only in the multivariate analysis. This approach may have some limitations. It may miss important confounding variables that are not significantly associated with the outcome in bivariate analysis but become significant when controlling for other factors. It can also lead to overfitting of the model. It is suggested to include variables based on theoretical importance and prior research evidence showing significant associations, regardless of their bivariate significance.

Minor points:

- In line 167, “Quantitative variables” may not be a suitable title, as it includes the explanation of conceptual framework of the variable.
- In the first sentence under ‘Quantitative variables’ section, the authors mentioned that the primary outcomes of interest in this study were two (diabetes and hypertension). I believe, authors missed obesity.
- In figure 1, for the full form of SDH, ‘of health’ might be missing.
- In line 184, Rstudio 1314 is mentioned. Is this correct?

**Results:**

- The distribution of participants were into urban poor, urban middle, and urban rich categories with the following proportions: Urban Rich: 49% (1694/3460), Urban Middle: 17% (590/3460), and Urban Poor: 34% (1176/3460). In urban populations, we typically expect to see a larger middle class and smaller proportions of the wealthiest group. However, this distribution shows a very large "rich" category and a relatively small "middle" category. The large proportion of "Urban Rich" (49%) is unusually high for most urban populations. Given that originally the wealth was categorized in wealth quintile with five categories, what was the logic behind for merging highest and fourth wealth quintile making urban rich and first and second into urban poor categories.
- In table 1, while in the total population, the sum of proportions make 100% vertically, the sum of proportions make 100% horizontally in urban rich, urban middle and urban poor, which makes table confusing.
- Please specify the variables that were considered in bivariate analysis but not included in the final model.
- As I mentioned earlier in statistical analysis, it is suggested to include variables based on theoretical importance and prior research evidence, regardless of their bivariate significance. Conducting sensitivity analyses to test the robustness of the results of multivariate analysis by including variables which were not significant in bivariate analysis might be helpful.

Minor points:

- Table 1 should be named as Table 2 because there is already table 1 in the method section.
- Add necessary footnotes in the tables1, 2, 3 and 4, for e.g., adding the variables adjusted in the adjusted model, specify where different analysis (chi-square or t-test) were conducted using markers such as * or #, full from of IQR, level of significance etc.
- Obesity can be replaced with BMI categories because obesity can have further categories, and replace ‘normal’ with ‘normal weight’.
- Instead of sufficient fruit and vegetable consumption, you may write fruit and vegetable consumption only and same for physical activity
- In table 2, 3 and 4, if it is wealth quintile, there should be five categories, however, there are only three categories.
- Consistency in tables: for e.g., in eduction status category secondary, please add (12 years) in all tables and add space between secondary and (12 years). Add the same number of digits after decimal in all cases.

**Discussion:**

- In the first paragraph, how relevant are references 18 and 19 in context of Nepalese urban population. Are there any references from Nepal?
- In fourth paragraph, the authors mentioned that the lower oxygen levels in mountainous regions may influence glucose metabolism, potentially reducing diabetes risk. This sentence needs reference, and what could be a potential mechanism behind it?
- Limitation is missing. I believe, there must be some limitations in the study.

Minor points:

- Please include line number in discussion and some parts of results.
- It may be better to rearrange the paragraphs based on the table numbers arranged in the results section, for e.g., discussing the results of table 1, followed by 2, 3 and 4, respectively, which might be easier for the readers to follow.
- In the second sentence of the first paragraph, the phrase “between the urban population” is not logical. The word “between” could be replaced with “within” the urban population or can be rephased as “among different groups within the urban population”.
