# Supplementary material for: Social determinants and risk factors associated with non-communicable diseases among urban population in Nepal: A comparative study of poor, middle and rich wealth categories of urban population using STEPS survey
Source: PLoS One. 2025 May 14;20(5):e0307622. doi: 10.1371/journal.pone.0307622 (PMC12077703; doi:10.1371/journal.pone.0307622)
Supplement: Attachment — Submitted filename: Response to Reviewers.docx [file pone.0307622.s006.docx]

**To,**

**The Editor,**

**PLOS ONE**

Subject: Response to the query from reviewers

Dear Editor,

Thank you for the evaluation from the reviewers on manuscript entitled on “Social determinants and risk factors linked to non-communicable diseases among urban populations in Nepal: A comparative analysis across poor, middle, and affluent wealth groups using the STEPS survey”.

We have responded all of the queries and comments of the reviewers. Please see below for our response to each of the comments/queries. We have also shared the revised manuscript in track changes for your review and approval.

Thank you very much for reviewing our study. If you need further clarification on this, please do not hesitate to contact us.

Yours sincerely,

Dr. Sampurna Kakchapati

Additional Editor Comments:

Editors' Feedback:

In this study, the authors examined the prevalence and contributing factors of non-communicable diseases (NCDs) within various wealth groups in Nepal.

This research introduces new data regarding the prevalence and relationship between NCDs and contextual factors, highlighting a crucial area of exploration. Nevertheless, the authors should address concerns regarding potential chance findings due to the extensive number of associations analyzed. To mitigate this, authors may consider applying a Bonferroni correction.

**Response:** Based on your suggestions, we had applied a Bonferroni correction in our analysis.

Despite these issues, the study offers valuable data and insights. I therefore suggest resubmitting the paper with revisions to address these points.

Typographical Errors

Abstract:

• Urban population Hilly region → Urban population from the Hilly region

**Response:** The text is revised as suggested. See page 2 and line no 41.

Introduction:

• Typographical errors:

o Socio-economic factors are associated with behavioral risk factors ..Remove "for" and mortality from NCDs.

**Response:** The text is revised and updated. See page 4 and line 75-77

• The phrases "Rapid urbanization, connection with vulnerable populations, and elevated NCDs risk" are unclear and challenging for readers. Please clarify what the authors intend to convey, particularly why the detection rate is increasing.

**Response:** The text is revised and updated as suggested. We have included the reasons for increment of the detection rate. Please see page 5, line 91-99.

• The statement "Urban residents face material constraints, limiting access to nutritious food, quality healthcare, and the ability to maintain healthy lifestyles" requires further elaboration. How do these constraints manifest?

**Response:** The further elaboration that Urban residents face material constraints, limiting access to nutritious food, quality healthcare, and the ability to maintain healthy lifestyles is added in the revised text. Please see page 5-6, line 111-119.

• The authors should expand their literature review to encompass the latest epidemiological knowledge, particularly in relation to the social determinants of NCDs, both in Nepal and internationally. This will help identify gaps in knowledge and strengthen the rationale for the study, as reviewers have also suggested.

**Response:** Based on your feedback and suggestions, we had expanded the introduction section using the latest epidemiological knowledge in regard to social determinants and NCDs nationally and globally.

Methodology:
• In the Site selection section, numerous data points lack citations.

**Response:** We have added the citations in the relevant sections as suggested in the revised manuscript.

• Clarify the term "weighted" in the context of multivariate logistic regression models.

**Response:** The term weighted is clarify in the methods section of manuscript. Please check page 10 & 11, line 181-184.

• Include the NHRC Ethical Approval Number.

**Response:** The NHRC Ethical Approval Number is added in the revised manuscript. Please see page 11, line 198-200.

Results:
• Given the extensive number of associations examined, there is a high likelihood of chance findings. Therefore, applying Bonferroni correction would be advisable.

**Response:** Based on the reviewers’ suggestions and comments, we had applied the Bonferroni corrections in the logistic regression. `

Discussion:
• The interpretation that "This finding underscores the potential vulnerability of all urban segments to NCDs and emphasizes the need for targeted interventions and policies across diverse urban populations" does not align with the results and previous statements. Please revise.

**Response:** This text is removed as suggested.

• The statement "This discrepancy may be attributed to hormonal differences, as well as lifestyle and behavioral factors more prevalent in males, such as higher rates of tobacco and alcohol consumption" should be directly linked to the study data on alcohol and smoking, rather than relying on citations.

**Response:** The recent STEP survey also shows male had higher prevalence of tobacco and alcohol consumption, since our study is using STEP Survey data, we can conclude that male had higher risk of smoking and alcohol consumption.

• Consider the application of Bonferroni correction, as only a few associations will likely remain significant, which should be the focus of the discussion.

**Response:** Yes, based on the reviewer’s comments and suggestions, we preformed the Bonferroni correction and as the results, only few associations was found to be significant.

Reviewers' comments:

Reviewer's Responses to Questions

**Comments to the Author**

Reviewer #1: Minor revisions to grammar suggested- occasional use of incorrect articles and verbs (a/and/the - was/were/is/are/had/have), extra spaces, incorrect plural versions of words, inconsistencies in capitalization and number formatting (i.e. 1,000 vs 1000), and comma-separated lists (line 202-203). Ensure that "sex" and "gender" are used consistently rather than interchangeably. Line numbering is lost after page 20.
**Response:** We have added line number after page 20 and revised the manuscript based in grammar suggestions.

Figure 1 is labeled as Figure 2. Table 1 could be made easier to understand with some formatting revisions. Ensure consistency in the use of the "STEP" acronym- in line 31, the approach is written as "STEP wise", while later in the paper, it becomes clear that a stepwise approach was used (see lines 115 & 123). Check the R Studio version reported in line 184. The definition of the salt consumption variable is not clear (unclear what "for first time" is referring to).
**Response:** Figure Number is revised as suggested. We ensure the correct use of STEP acronyms in the revised manuscript. The acronym for STEPwise approach to NCD risk factor surveillance is added as suggested. See Page 7, Line 31-32. R Studio version is added in the revised manuscript.

The discussion section could benefit from expanded explanations of contradictory findings. In some areas of the methods and results section, it is not fully clear whether the authors have controlled for age in the logistic regression models (for example, within the hypertension outcome between students and homemakers). Clarification of the models and adjustments to models for each of the two main outcomes would increase understanding of results.
**Response:** Based on the feedback of first reviewers, we had performed the Bonferroni correction and revised the analysis and table. Now the regression analysis and Tables are updated. During the Bonferroni correction, we also adjust the age and homemakers.

Ensure correct links in reference list- i.e. reference #1 links to a Google Search, rather than the article.
**Response**: Added new reference list for reference 1 based on your suggestions.

Overall assessment: Minor Revisions

Reviewer #2: Dear Editor and Valued authors

The manuscript “PONE-D-24-27355” entitled “Social determinants and risk factors associated with non-communicable diseases among urban population in Nepal: A comparative study of poor, middle and rich wealth categories of urban population using STEP survey” in itself is well written and represents a comprehensive study in relation to the risk factors for non-communicable diseases (NCDs). While scanning the manuscript thoroughly, it is understood that authors have clearly mentioned the methodological design with multistage stratified technique. One of the crucial parts of this paper is noticed that it has incorporated almost all socio-demographical parameters which might contribute as risk factors for NCDs. However, some suggestions are provided below which would enable further strength of the manuscript and imply its importance in public health domain. This paper can be considered provided the below mentioned aspects are satisfactorily addressed. In a nutshell, a minor revision is required.

Suggestions/Comments:

1. This manuscript is silent regarding the hypothesis of the study which is deemed pertinent in such kind of public health related analytical study. It would be fine to mention the hypothesis briefly in the last paragraph of the “Introduction” section. Please consider it in the revised MS.

**Response:** We had added the hypothesis of the study in the introduction section of revised manuscript. Please see Page 7, lines 114-116.

2. As mentioned in Table 1, what does the term “average day” indicate? Please specify.

**Response:** We had specified the average day in the revised manuscript.

3. The variable “24 hours salt consumption” seems to be poorly defined (Table 1). Please redefine it.

**Response:** We had defined the “24 hours salt consumption” in the revised manuscript along with citations.

4. The term “Terai” used in the MS should be mentioned as “Tarai” as it is a typical Nepali naming and should look like an original one.

**Response:** The text is replaced in the updated manuscript.

5. In Methods section or elsewhere, a country map of Nepal indicating the proportion of study participants representing the provincial location and ecological belt would be worth incorporating. If possible, the dominant associated risk factors for NCDs be mentioned with specific notation (i.e., with different color code or same color with decreasing intensity) in the specific ecological and political zone of the country. This would provide readers an overall picture of the entire study.

**Response:** We can include the country map of Nepal as suggested. However, due to tables and figures limitations for the manuscript submission, it is hard to add map of Nepal. Please provide your suggestions.

6. Minor typo errors need to be corrected throughout the MS.
**Response:** Revised and updated.

Regards,

Reviewer #3: Comments to the authors:
Thank you for the opportunity to review this paper. This manuscript specifically compared the social determinants, NCD risks and prevalence among different wealth categories and determined the factors associated with different NCDs among urban population in Nepal. Given the limited epidemiological evidence on the risk factors and determinants of NCDs in low- and middle-income countries, including Nepal, this study had tried to address a growing concern of the impact of increasing urbanization and how socioeconomic status affects NCD risk and prevalence within urban settings using the recent data from a 2019 STEPS survey from Nepal. However, I have several comments and suggestions which might be helpful to improve the manuscript.

Abstract:
Methods:
• Study design is not mentioned

**Response:** We have added the study design in the methods section of manuscript.

• You can specify the age group of the participants.
**Response:** The age group of the participants is added.

Results: You may include some important result data from table
**Response:** We had included important result data from table as suggestions.

Conclusion: Recommendations are quite general and are not specific based on the study findings. I would suggest making them more specific that align with the study findings.
**Response:** Recommendations is made more specific and align with the study findings in the revised manuscript.

Minor points:
• In line 32, you can add acronym for STEPwise approach to NCD risk factor surveillance

**Response:** The acronym for STEPwise approach to NCD risk factor surveillance is added as suggested. See Page 7, Line 31-32

• In line 41, spell out AOR when it appeared for the first time in the abstract also.
**Response:** We had spell out in the revised manuscript. Please see page 2, line 41.

Introduction:
• Please cite more previous studies from Nepal to explain why this study is important among urban population in Nepal.

**Response:** We are cited the studies from Nepal and added why this study is important among urban population in Nepal. Please see Page 6, line 111-129.

• In the last paragraph, the authors mentioned “this study aims to inform effective strategies that mitigate the burden of NCDs and enhance the overall well-being of the urban population in Nepal.” Was this also the study objective and how was this done from this study?

**Response:** The sentence may be linked with objective of the study. As findings of the study helps to inform effective strategies that mitigate the burden of NCDs and enhance the overall well-being of the urban population as the findings is related to urban population and social stratifies that is related to urban population.

• Line 74: grammar, ‘for’ after behavioral risk factors may be removed.

**Response:** Removed as suggested

• Line 94-95: add citation to the sentence ‘The dynamics of urban health in Nepal is associated with a complex and interlinked range of wider determinants’

**Response:** We had added the citation as suggested.

• Lines 99-100: add citation to the line “Furthermore, increasing rates….complexity to public health challenges”.

**Response:** We had revised this text and added the citations where relevant.

• Lines 102-104: add citation to the line “the co-influence of socio-economic factors…..social determinants and health outcomes.
**Response:** We had added the citation as suggested.

Methods:
Study design and sampling size:
• Please provide reference of the STEPS survey.

**Response:** We had added the reference of STEPS survey as suggested.

• The section title mentions "sampling size", but no specific information on sample size calculation is provided. Sampling size may not be the right word, instead sampling technique might be better.

**Response:** We had removed the sampling size in the revised manuscript.

• Under ‘Study design and sampling size’, information about data collection and survey instrument is also mentioned. You may create a separate section for this, and you can explain more about survey questions.

**Response:** We had revised the sections as suggested.

Study setting:
• It might be better to include ‘study setting’ first, followed by study design and sampling. Study setting can help establish the context of the study design and sampling methods.

**Response:** We had revised the sections as suggested. We include study setting first followed by study design.

• While the description of Nepal's geography is informative in study setting, the description is a bit long compared to other sections. It would be better to make it shorter and link it with the study design and sampling in next paragraph.

**Response:** Based your suggestions, we have summaries the Nepal geography in the study setting.

• There is an error in the urbanization statistics. Line 144 "66% of the population living in urban areas in 2021 compared to just 23% in 2021" uses the same year twice, which does not make sense. The years should be different for this comparison.

**Response:** We had revised the text as suggested.

• Please provide a citation for the urbanization and population distribution statistics provided in the study setting, particularly if they are not from the STEPS survey data.

**Response:** We had added the citation as suggested.

Participant:
• First sentence has already been mentioned in the section ‘study design and sampling size section’ and has been repeated here.

**Response:** We had removed the text as suggested.

• Authors have mentioned that they included 3,460 individuals from urban areas in the analysis. It is not clear how 3,460 participants for this study were selected from the total population (6,475). The definition of urban area is not clear in the study and should be clearly defined.

**Response:** The selection of 3,460 participants was based on a subset of individuals who were classified as residing in urban areas from the total population of 6,475 participants in the original dataset. The selection was guided by predefined criteria for urban residence, ensuring that only those participants who live in metropolitan cities, sub-metropolitan cities, municipalities were considered as urban population and participants from rural municipalizes we considered as rural population. This information is added in the revised text. Please see Page 7-8, line 154-169.

Variable definitions:
• How was salt consumption measured in the STEPS survey and how was this defined in this study? Given that salt is necessary nutrient for our body in smaller amount and over consumption could be harmful, did the participants respond as ‘yes’ or ‘no’ for salt consumption in the past 24 hours as shown in table 1?

**Response:** We had added the salt consumption measured in the STEP survey in the Table 1. We had also provided the citation for salt consumption.

• Please check the definition of pre-hypertension, hypertension, and pre-diabetes.

**Response:** Yes, we check the definition of pre-hypertension, hypertension, and pre-diabetes.

Statistical analysis:

• Three categories were created from 5 wealth quintiles creating unevenly distributed categories. What was the reason for authors to merge categories? Did you try to analyze data using five original categories (5 quintiles)?

**Response:** We had analyses the data using five original categories (5 quintiles). However, we categories for three groups as our study are more focus on urban poor and urban rich.

• In the analysis, authors mentioned that they included variables one at a time in the univariate analysis and then including significant variables only in the multivariate analysis. This approach may have some limitations. It may miss important confounding variables that are not significantly associated with the outcome in bivariate analysis but become significant when controlling for other factors. It can also lead to overfitting of the model. It is suggested to include variables based on theoretical importance and prior research evidence showing significant associations, regardless of their bivariate significance.
**Response:** Based on the feedback of first reviewers, we had performed the Bonferroni correction for regression analyses and revised the analysis and table. Now the regression analysis and Tables are updated.

Minor points:
• In line 167, “Quantitative variables” may not be a suitable title, as it includes the explanation of conceptual framework of the variable.

**Response:** Revised to make it as study variables.

• In the first sentence under ‘Quantitative variables’ section, the authors mentioned that the primary outcomes of interest in this study were two (diabetes and hypertension). I believe, authors missed obesity.

**Response:** We had added the obesity in the study variable section.

• In figure 1, for the full form of SDH, ‘of health’ might be missing.

**Response:** We had full form of SDH. Page 10, Line 174-175.

• In line 184, Rstudio 1314 is mentioned. Is this correct?

**Response:** We have used R version 4.2.0

Results:
• The distribution of participants were into urban poor, urban middle, and urban rich categories with the following proportions: Urban Rich: 49% (1694/3460), Urban Middle: 17% (590/3460), and Urban Poor: 34% (1176/3460). In urban populations, we typically expect to see a larger middle class and smaller proportions of the wealthiest group. However, this distribution shows a very large "rich" category and a relatively small "middle" category. The large proportion of "Urban Rich" (49%) is unusually high for most urban populations. Given that originally the wealth was categorized in wealth quintile with five categories, what was the logic behind for merging highest and fourth wealth quintile making urban rich and first and second into urban poor categories.

**Response:** In response to the reviewer's comment regarding the merging of wealth quintiles into three categories (Urban Poor, Urban Middle, and Urban Rich), the rationale behind this decision was based on both statistical and analytical considerations. First, the distribution of participants across the original five wealth quintiles revealed significant disparities in sample sizes. By merging the first and second quintiles into the "Urban Poor" category, and the fourth and fifth quintiles into the "Urban Rich" category, we were able to create more balanced groups, ensuring adequate sample sizes for meaningful statistical analysis and comparison. Second, the primary focus of our analysis was to examine the extremes of the wealth distribution, particularly the richest and poorest segments of the urban population, in order to better understand the social determinants and risk factors for NCDs. The middle quintile was maintained as a distinct category to preserve analytical clarity and allow for direct comparisons between these wealth groups. That said, we are open to further discussions or reconsideration of the five original wealth quintiles if necessary for more granular analysis.

• In table 1, while in the total population, the sum of proportions make 100% vertically, the sum of proportions make 100% horizontally in urban rich, urban middle and urban poor, which makes table confusing.

**Response:** We have revised the table as suggested. Now the sum of proportion makes 100%.

• Please specify the variables that were considered in bivariate analysis but not included in the final model.

**Response:** We had specified the section as suggested. Please see page 11, line 183-184.

• As I mentioned earlier in statistical analysis, it is suggested to include variables based on theoretical importance and prior research evidence, regardless of their bivariate significance. Conducting sensitivity analyses to test the robustness of the results of multivariate analysis by including variables which were not significant in bivariate analysis might be helpful.

**Response:** Based on the feedback of first reviewers, we had performed the Bonferroni correction for regression analyses and revised the analysis and table. Now the regression analysis and Tables are updated.

Minor points:

• Table 1 should be named as Table 2 because there is already table 1 in the method section.

**Response:** We had revised the Table number as suggested.

• Add necessary footnotes in the tables1, 2, 3 and 4, for eg. Adding the variables adjusted in the adjusted model, specify where different analysis (chi-square or t-test) were conducted using markers such as * or #, full of IQR, level of significance etc.

**Response:** We had added the footnotes of t-test in the table.

• Obesity can be replaced with BMI categories because obesity can have further categories, and replace ‘normal’ with ‘normal weight’.

**Response:** We had replaced the obesity with BMI Categories in the revised manuscript.

• Instead of sufficient fruit and vegetable consumption, you may write fruit and vegetable consumption only and same for physical activity

**Response:** We respectfully disagree with the suggestion to remove "sufficient" from "fruit and vegetable consumption" and "physical activity." The term sufficient fruit and vegetable consumption was based on the operational definition of the study (Table 1).

• In table 2, 3 and 4, if it is wealth quintile, there should be five categories, however, there are only three categories.

**Response:** In response to the reviewer's comment regarding the merging of wealth quintiles into three categories (Urban Poor, Urban Middle, and Urban Rich), the rationale behind this decision was based on both statistical and analytical considerations. First, the distribution of participants across the original five wealth quintiles revealed significant disparities in sample sizes. By merging the first and second quintiles into the "Urban Poor" category, and the fourth and fifth quintiles into the "Urban Rich" category, we were able to create more balanced groups, ensuring adequate sample sizes for meaningful statistical analysis and comparison. However, we are open to further discussions or reconsideration of the five original wealth quintiles if necessary for more granular analysis.

• Consistency in tables: for eg., in eduction status category secondary, please add (12 years) in all tables and add space between secondary and (12 years). Add the same number of digits after decimal in all cases.

**Response:** Removed and add the definition of education in operational definition. Please check the Table 1.

Discussion:
• In the first paragraph, how relevant are references 18 and 19 in context of Nepalese urban population. Are there any references from Nepal?

**Response:** We have revised the references as suggested.

• In fourth paragraph, the authors mentioned that the lower oxygen levels in mountainous regions may influence glucose metabolism, potentially reducing diabetes risk. This sentence needs reference, and what could be a potential mechanism behind it?

**Response**: We had removed the text based on reanalysis the data.

• Limitation is missing. I believe, there must be some limitations in the study.

**Response:** We had included the limitations of the study in the revised manuscript. Please see page 28-29, line 396-402.

Minor points:
• Please include line number in discussion and some parts of results.

**Response**: We had included the line number in discussion and some parts of results.

• It may be better to rearrange the paragraphs based on the table numbers arranged in the results section, for eg., discussing the results of table 1, followed by 2, 3 and 4, respectively, which might be easier for the readers to follow.
**Response:** We have revised the arrangement of table number in the revise manuscript.

• In the second sentence of the first paragraph, the phrase “between the urban population” is not logical.

The word “between” could be replaced with “within” the urban population or can be rephased as “among different groups within the urban population”.

**Response:** The sentence is replaced as suggested.
