# Supplementary material for: Social determinants and risk factors associated with non-communicable diseases among urban population in Nepal: A comparative study of poor, middle and rich wealth categories of urban population using STEPS survey
Source: PLoS One. 2025 May 14;20(5):e0307622. doi: 10.1371/journal.pone.0307622 (PMC12077703; doi:10.1371/journal.pone.0307622)
Supplement: Attachment — Submitted filename: Comments_to_authors_Manuscript_PONE-D-24-27355_declet_1.docx [file pone.0307622.s007.docx]

**Comments to the authors:**

Overall, the authors have responded most of the reviewers’ comments and suggestions. However, some points need futher clarification and some minor corrections, which can improve the quality of the the manuscript. If the comments below are addressed appropriately, the manuscript could be accepted.

1. Adding “cross sectional” study design would be better, as it would strengthen the abstract.
2. The sentence “The significant factors ….higher odds of obesity (AOR=1.01, 95% CI=1.01-1.02).” in lines 40-45 in abstract is lengthy and included several associations, making it harder to follow. Breaking it into shorter sentences can improve clarity.
3. In line 50-51, ‘Being obesity should be ‘being obese’ and obese should be replaced with ‘overweight’
4. In line 81, replace ‘in seek for’ with ‘seeking for’ or ‘in search of’.
5. Line 124-126, suggest that the country is among the fastest urbanizing in Asia. However, this does not align with the provided data which suggest the slow pace of urbanization from 62.9% in 2011 to 66% in 2021. Please check the data again and revise the sentence accordingly.
6. The sentence in line 146 ‘Data for the study was obtained from STEP survey’ has already appeared in line 133 and can be removed.
7. Though the authors responded that they checked the definitions, the definition of pre-hypertension is still identical to that of hypertension. Please correct the definition. If this definition was used to categorize the participants, I suppose the number of participants falling into the pre-hypertension group might change after the correction, which could impact the results. Please reference the standard guidelines used to define diabetes and hypertension.
8. The authors highlighted the need to balance sample sizes across categories by merging quintiles into three wealth groups, which is a legitimate statistical consideration. However, disproportionately large "Urban Rich" category (49%) may not represent a true "rich" population but may include a mixed wealth group. Important differences between the original quintiles may have been masked by the merging the two groups, especially if health behaviors or outcomes differ significantly between groups. Therefore, please provide the original quintile sample sizes, which would clarify whether the merging was truly necessary or justified to balance group sizes or improving analysis. If combining quintiles is necessary, the authors should provide evidence (e.g., showing similar demographic or health characteristics and outcomes between combined groups), and explain and clearify to justify the merging. This would strengthen the logic for merging. Additionally, this categorization could be one of the study limitations.
9. In line 164, ‘….were two common NCDs: obesity, hypertension and diabetes’ – ‘Two’ should be replaced with three.
10. The sentence in lines 189 and 190 ‘Variables that were considered in bivariate analysis but not included in the final model’ is not clear. Clarifying the variables adjusted for in the multivariable models for each outcome would improve understand the results. This information can also be included in the footnotes of the relevant tables.
11. NHRC should be spelled out at its first mention in line 198, instead of line 199.
12. In line 205, Table 1 should be replaced with Table 2.
13. In line 205, it is written that “Table 1 shows the comparison of risk behaviors that include socio demographic factors, behavioral factors biological factors and NCD among poor, middle and rich wealth categories of urban population.” Socio-demographic factors are not risk behaviors. Please revise accordingly.
14. Given the results are presented as odds ratio in table 3, 4 and 5, the inclusion of ‘t-test’ in the footnotes in those tables is confusing.
