# Supplementary material for: Social determinants and risk factors associated with non-communicable diseases among urban population in Nepal: A comparative study of poor, middle and rich wealth categories of urban population using STEPS survey
Source: PLoS One. 2025 May 14;20(5):e0307622. doi: 10.1371/journal.pone.0307622 (PMC12077703; doi:10.1371/journal.pone.0307622)
Supplement: Attachment — Submitted filename: Comments_to_authors_Manuscript_PONE-D-24-27355_declet_2.docx [file pone.0307622.s009.docx]

**Comments to the authors:**

The authors have made substantial efforts to address most of the previous comments. For further clarity of the manuscript and to strengthen the methodology, I would like to suggest a few minor revisions. The manuscript can be considered for acceptance once all these minor revisions are made.

1. While the definition of pre-hypertension has been corrected in Table 1 and the authors responded that they use standard guidelines in the response letter, I would suggest adding references to these guidelines in Table 1 of the manuscript as well, as multiple definitions exist. This will ensure clarity for the readers.
2. In the conceptual framework, the link between structural factors and intermediary factors is missing, which is important for understanding the pathways leading to inequities. A minor suggestion is to add an arrow to illustrate these relationships, making the framework clearer for readers. Additionally, was this framework developed based on any existing models or established frameworks? If so, please provide appropriate reference in the manuscript. A brief explanation of the model in the manuscript would make readers easy to understand the framework.
3. The authors have provided the original quintile sample sizes and clarified that the 'Urban Poor' category is disproportionately large in the revised manuscript. However, the justification for merging these groups should be clearly presented in the main text, rather than only in the response to reviewers, to ensure that all readers understand the reasoning behind the decision. Additionally, since the authors mentioned that further analyses were conducted to support the merging, these analyses could be included in the manuscript, preferably as a supplementary table. Without this supporting evidence or explanation in the text, I still feel that it remains unclear for readers whether the merging was justified.
