# Supplementary material for: Social determinants and risk factors associated with non-communicable diseases among urban population in Nepal: A comparative study of poor, middle and rich wealth categories of urban population using STEPS survey
Source: PLoS One. 2025 May 14;20(5):e0307622. doi: 10.1371/journal.pone.0307622 (PMC12077703; doi:10.1371/journal.pone.0307622)
Supplement: Attachment — Submitted filename: Response_To_Reviewers_auresp_3.docx [file pone.0307622.s010.docx]

Date: January 11, 2025

To,

The Editor, Reviewers,

PLOS ONE

**Subject: Addressing Editor/Reviewer Comments for [PONE-D-24-27355]**

Dear Editor, Reviewers,

Many thanks for your valuable and constructive feedback on our manuscript. We have carefully considered all of your comments and have made the necessary revisions to address the issues raised. We believe these changes have significantly improved the quality of the paper.

Below, we have outlined our responses to your comments, with the actions we have taken to address each point highlighted in italics. We hope these revisions further enhance the quality of our work.

Thank you again for your time and consideration.

Kind regards,

Authors of **PONE-D-24-27355**

**Comments to the authors:**

Overall, the authors have responded most of the reviewers’ comments and suggestions. However, some points need further clarification and some minor corrections, which can improve the quality of the manuscript. If the comments below are addressed appropriately, the manuscript could be accepted.

1. Adding “cross sectional” study design would be better, as it would strengthen the abstract.

***Response****: We had added cross sectional study design in the abstract. The revised text is added on line 31, page 2.*

2. The sentence “The significant factors ….higher odds of obesity (AOR=1.01, 95% CI=1.01-1.02).” in lines 40-45 in abstract is lengthy and included several associations, making it harder to follow. Breaking it into shorter sentences can improve clarity.

***Response****: We had broken the sentence into shorter sentences. The revised text is added on line 43-47, page 2-3.*

3. In line 50-51, ‘Being obesity should be ‘being obese’ and obese should be replaced with ‘overweight’

***Response:*** *Thank you for your valuable feedback. We have addressed your suggestion and replaced "being obesity" with "being obese" and revised "obese" to "overweight" at page 3, line 54.*

4. In line 81, replace ‘in seek for’ with ‘seeking for’ or ‘in search of’.

***Response:*** *Thank you for your feedback. We have replaced "in seek for" with "in search of" in as suggested in line 86, page 4 .*

5. Line 124-126, suggest that the country is among the fastest urbanizing in Asia. However, this does not align with the provided data which suggest the slow pace of urbanization from 62.9% in 2011 to 66% in 2021. Please check the data again and revise the sentence accordingly.

***Response:*** *Thank you for pointing this out. Upon review, we have confirmed the data and revised the sentence accordingly. The revised text highlights the significant increase in urbanization between 2001 and 2011, followed by a slower pace of growth from 2011 to 2021. This adjustment aligns the interpretation with the provided data. Please see page 6, line 130-133.*

6. The sentence in line 146 ‘Data for the study was obtained from STEP survey’ has already appeared in line 133 and can be removed.

***Response:*** *Removed as suggested.*

7. Though the authors responded that they checked the definitions, the definition of pre-hypertension is still identical to that of hypertension. Please correct the definition. If this definition was used to categorize the participants, I suppose the number of participants falling into the pre-hypertension group might change after the correction, which could impact the results. Please reference the standard guidelines used to define diabetes and hypertension.

***Response:*** *Thank you for your valuable feedback. We apologize for the confusion regarding the definitions of pre-hypertension and hypertension. We have rectified the definition of pre-hypertension to align with standard clinical guidelines. Specifically, pre-hypertension is now defined as having an average systolic BP greater than or equal to 120 mmHg but less than 140 mmHg, or an average diastolic BP greater than or equal to 80 mmHg but less than 90 mmHg. This correction has been made, and we expect the number of participants categorized as pre-hypertensive to change accordingly, which may impact the results. We have referenced the standard guidelines for hypertension and pre-hypertension as provided by the American College of Cardiology (ACC) and the American Heart Association (AHA) in the revised manuscript. The revised text is added in Table 1.*

8. The authors highlighted the need to balance sample sizes across categories by merging quintiles into three wealth groups, which is a legitimate statistical consideration. However, disproportionately large "Urban Rich" category (49%) may not represent a true "rich" population but may include a mixed wealth group. Important differences between the original quintiles may have been masked by the merging the two groups, especially if health behaviors or outcomes differ significantly between groups. Therefore, please provide the original quintile sample sizes, which would clarify whether the merging was truly necessary or justified to balance group sizes or improving analysis. If combining quintiles is necessary, the authors should provide evidence (e.g., showing similar demographic or health characteristics and outcomes between combined groups), and explain and clarify to justify the merging. This would strengthen the logic for merging. Additionally, this categorization could be one of the study limitations.

*Response: Thank you for your insightful comment. We have carefully checked the data regarding the "Urban Poor" category and found that it includes a disproportionately large proportion of participants (49%), not the "Urban Rich" as originally suggested. This suggests that the "Urban Poor" category may represent a mixed wealth group rather than solely those classified as poor. We have now provided the original sample sizes for each quintile to clarify whether merging the groups was necessary to balance sample sizes or improve analysis. The sample sizes for each quintile are as follows: Poorest quintile: 1014, Second quintile: 680, Third quintile: 590, Fourth quintile: 550 and Fifth quintile: 626*

*Additionally, to justify the merging of the "Urban Poor" and "Urban Middle" groups, we conducted further analyses comparing the demographic and health characteristics between the combined groups. These analyses revealed that health behaviors and outcomes were relatively similar across the combined groups, which supports our decision to merge them for analytical purposes. We have added these sample size of each quintile in the Table 1 with the revised definition of urban rich, urban middle and urban poor.*

9. In line 164, ‘….were two common NCDs: obesity, hypertension and diabetes’ – ‘Two’ should be replaced with three.

***Response:*** *Yes, we had replaced two with three common NCDs in line 174.*

10. The sentence in lines 189 and 190 ‘Variables that were considered in bivariate analysis but not included in the final model’ is not clear. Clarifying the variables adjusted for in the multivariable models for each outcome would improve understand the results. This information can also be included in the footnotes of the relevant tables.

***Response****: Thank you for your valuable feedback. We agree that the sentence in lines 189 and 190 could be clearer. To improve clarity, we have revised the sentence to explicitly state the variables that were adjusted for in the multivariable models for each outcome at Page 12, 199-200.*

11. NHRC should be spelled out at its first mention in line 198, instead of line 199.

***Response:*** *We had spelled NHRC in first mention*.

12. In line 205, Table 1 should be replaced with Table 2.

***Response:*** *Yes, we replace Table 1 with Table 2.*

13. In line 205, it is written that “Table 1 shows the comparison of risk behaviors that include socio demographic factors, behavioral factors biological factors and NCD among poor, middle and rich wealth categories of urban population.” Socio-demographic factors are not risk behaviors. Please revise accordingly.

***Response:*** *Yes, we have revised the text accordingly at line 218-219, page 13.*

14. Given the results are presented as odds ratio in table 3, 4 and 5, the inclusion of ‘t-test’ in the footnotes in those tables is confusing.

***Response****: Thank you for pointing out the inconsistency regarding the use of the t-test in the footnotes. We have used the t-test for the results presented in Table 2, where we compare the means between groups. However, we have removed the mention of the t-test in the footnotes of Tables 3, 4, and 5, as these tables present results in terms of odds ratios, which are derived from logistic regression rather than t-tests. We appreciate your attention to this detail, and the revised footnotes should now accurately reflect the statistical methods used in each table.*
